# Spatiotemporal Immunomodulation and Biphasic Osteo‐Vascular Aligned Electrospun Membrane for Diabetic Periosteum Regeneration

**DOI:** 10.1002/advs.202302874

**Published:** 2023-11-16

**Authors:** Yusen Qiao, Lei Yu, Peng Yang, Miao Chen, Haifu Sun, Lingjie Wang, Bangzhao Wu, Chun‐do Oh, Huilin Yang, Jiaxiang Bai, Dechun Geng

**Affiliations:** ^1^ Department of Orthopedics The First Affiliated Hospital of Soochow University 188 Shizi Road Suzhou Jiangsu 215006 China; ^2^ Department of Orthopedic Surgery Rush University Medical Center Chicago IL 60612 USA; ^3^ Department of Orthopedics, Centre for Leading Medicine and Advanced Technologies of IHM, The First Affiliated Hospital of USTC, Division of Life Sciences and Medicine University of Science and Technology of China Hefei 230022 China; ^4^ National Center for Translational Medicine (Shanghai) SHU Branch Shanghai University Shanghai China

**Keywords:** calvarial periosteal defects, diabetes mellitus, electrospinning, immunomodulation, liposomes

## Abstract

Under diabetic conditions, blood glucose fluctuations and exacerbated immunopathological inflammatory environments pose significant challenges to periosteal regenerative repair strategies. Responsive immune regulation in damaged tissues is critical for the immune microenvironment, osteogenesis, and angiogenesis stabilization. Considering the high‐glucose microenvironment of such acute injury sites, a functional glucose‐responsive immunomodulation‐assisted periosteal regeneration composite material—PLA（Polylactic Acid）/COLI(Collagen I）/Lipo(Liposome）‐APY29 (PCLA)—is constructed. Aside from stimulating osteogenic differentiation, owing to the presence of surface self‐assembled type I collagen in the scaffolds, PCLA can directly respond to focal area high‐glucose microenvironments. The PCLA scaffolds trigger the release of APY29‐loaded liposomes, shifting the macrophages toward the M2 phenotype, inhibiting the release of inflammatory cytokines, improving the bone immune microenvironment, and promoting osteogenic differentiation and angiogenesis. Bioinformatics analyses show that PCLA enhances bone repair by inhibiting the inflammatory signal pathway regulating the polarization direction and promoting osteogenic and angiogenic gene expression. In the calvarial periosteal defect model of diabetic rats, PCLA scaffolds induce M2 macrophage polarization and improve the inflammatory microenvironment, significantly accelerating periosteal repair. Overall, the PCLA scaffold material regulates immunity in fluctuating high‐glucose inflammatory microenvironments, achieves relatively stable and favorable osteogenic microenvironments, and facilitates the effective design of functionalized biomaterials for bone regeneration therapy in patients with diabetes.

## Graphical abstract

Multifunctional PLA/COLI/Lipo‐APY29 (PCLA) is prepared for diabetic rat cranial defects. Glucose‐responsive 4‐fluorophenyl boric acid (FPBA) moiety prior to encapsulation with APY29 endows the electrospun scaffolds with immunomodulation ability, and the self‐assembled collagen enhances the osteogenic capacity. Therefore, the functionalized electrospun scaffolds can regulate the immune microenvironment at the defect site to enhance the degree of vascularized bone regeneration and promote periosteum healing.

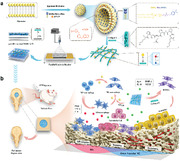

## 1 Introduction

Diabetes mellitus (DM) is a long‐term metabolic disorder that affected ≈425 million people globally in 2017, and the number is estimated to surpass 629 million by 2035.^[^ ^1^ ^]^ In addition to well‐known macrovascular and microvascular complications, such as strokes, retinopathy, neuropathy, and nephropathy, patients suffering from DM have a higher risk of fractures than healthy individuals due to their disordered glucose metabolism and increased basal inflammatory levels in vivo. Indeed, diabetes increases the risk of fractures by ≈20–300%.^[^ ^2^ ^]^ Usually, the periosteum contributes to >70% of the bone and cartilage formation in the early stages of fracture healing. However, in the DM microenvironment, chronic inflammation and excessive accumulation of advanced glycation end products, as well as the presence of reactive oxygen species, may damage mesenchymal stem cells and factors such as growth factors. This would lead to the weakening of osteogenic differentiation, reduced angiogenesis, and the aggravation of poor periosteal healing, ultimately resulting in a marked rise of bone defects.^[^ ^3^ ^]^ It is therefore imperative to re‐examine the strategy of periosteal regeneration for patients with DM.

Currently, many methods are available to improve bone healing, including drugs, growth factors, ultrasound, lasers, and pulsed electromagnetic fields.^[^ ^4^ ^]^ However, insufficient bone formation and the inability to accurately and controllably release growth factors or drugs limit their practical applications.^[^ ^5^ ^]^ In addition, traditional bone repair methods focus mainly on optimizing osteogenesis, and they generally employ inert materials to avoid immune rejection, ignoring the essential functions of the immune system in tissue repair and regeneration.^[^ ^6^ ^]^ Ignoring immune regulation and focusing only on osteoinduction often results in failure to adapt to the pathological microenvironment.^[^ ^7^ ^]^ As the primary defense of the innate system, macrophages possess an anti‐inflammatory and therapeutic phenotype (M2) that relieves inflammation and generates a beneficial immunological microenvironment for the regeneration of bone. However, the DM microenvironment is usually accompanied by fluctuations in blood glucose levels, which further prolong the local residence time of pro‐inflammatory phenotype (M1) macrophages and significantly delay the production of M2 macrophages, further aggravating inflammation and leading to damage during bone repair. The dynamic transition from M1 to M2 phenotype therefore cannot be successfully completed under chronic inflammatory conditions.^[^ ^8^ ^]^ It is necessary to rationally design bone repair materials that can dynamically respond to changes in blood glucose levels, and that possess multiple functions, such as immune regulation and bone induction. Such materials should also be able to accurately coordinate and arrange various biological events during the bone healing process.

Glucose‐responsive drug delivery systems based on micro/nanoparticles are promising therapeutic drug delivery strategies.^[^ ^9^ ^]^ For example, liposomes have been imparted with stimuli‐response characteristics that can trigger the complete release of encapsulated drug molecules. When the liposomal surface membrane becomes porous, the contents of the liposome begin to leak, which, in turn, leads to a further reduction in the membrane tension and eventual rupture. In the DM microenvironment, the high glucose concentration can promote endogenous stimulation and modify the structural state of the liposomes. Phenylboronic acid (PBA) is a common glucose‐responsive component that possesses good structural stability and flexibility, in addition to being easily prepared at a low price. As such, PBA can combine with the hydroxyl groups at the 1,2 or 1,3 positions of glucose to form a dynamic borate structure.^[^ ^10^ ^]^ This reaction imparts a further negative charge on the PBA, thereby indicating that the PBA‐stimulated surface strategy has the potential to modify liposomes, which are positively charged.^[^ ^11^ ^]^ However, the stability and dynamic exchange characteristics of the borates formed by this modification approach are poor due to the various boric acid structures formed at different acidities, wherein the pKa of PBA ranges from 7.8 to 8.6. More specifically, in a physiological environment (pH 7.4 < pKa), PBA transforms into an uncharged planar structure that has difficulty reacting with hydroxyl groups. As a result, the glucose borate becomes unstable and is easily hydrolyzed, resulting in low‐glucose reactivity.^[^ ^12^ ^]^ To address this issue, an electrophilic group was introduced into PBA to generate 4‐fluorophenyl boric acid (FPBA) with a reduced pKa of 7.2.^[^ ^13^ ^]^ Interestingly, using this strategy, the negative charge resulting from the combination of glucose and FPBA can cause FPBA to change from hydrophobic to hydrophilic. It was therefore envisaged that FPBA could be used to modify the surfaces of liposomes via the glucose response. More specifically, any glucose present should conjugate with the FPBA units on the liposomal membrane, causing the membrane to expand through structural transformation and the loaded drug or factor to be released.

Recently, electrospinning has been widely used to replicate the physiological microenvironment that guides cell fate and regulates tissue regeneration because of its similarity to the natural extracellular matrix (ECM). Since functional electrospinning can autonomously respond to external stimuli and actively fine‐tune the characteristics or plan the delivery of therapeutic compounds, it is considered a good candidate for periosteal regeneration.^[^ ^14^ ^]^ Regulation of the local immunological microenvironment and in‐situ bone‐forming osteoblastic differentiation of mesenchymal stem cells are critical for diabetic bone deficits.

We herein consider loading liposomes with a specific immunomodulator (APY29) and subjecting this species to subsequent spinning in combination with type I collagen (COLI). Type I collagen is the main organic component of ECM in bone, contributing ≈90% of the total organic components of the bone matrix.^[^ ^15^ ^]^ In addition, type I collagen can promote osteogenic differentiation and mineralization of bone marrow stromal cells and human adipose stem cells.^[^ ^16^ ^]^ Multiple studies have demonstrated that Gingistat, a collagen scaffold, supports the distribution and osteogenic differentiation of MSCs,^[^ ^17^ ^]^ has extensive neo‐osteoid formation in the implant region, successfully cures critical dimensional defects in rat femurs and pig tibias, and plays an important role in bone tissue engineering.^[^ ^18^ ^]^

**Scheme 1:**
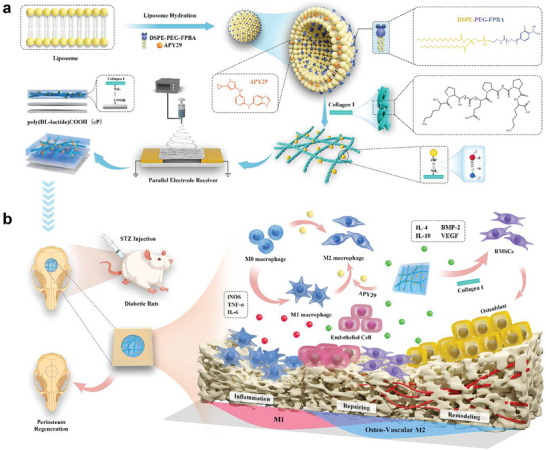
Schematic illustration of the preparation process used to obtain the multifunctional PLA/COLI/Lipo‐APY29 (PCLA). a) Initially, liposomes are modified with the glucose‐responsive 4‐fluorophenyl boric acid (FPBA) moiety prior to encapsulation with a specific immunomodulator, namely APY29. Second, COLI‐bearing glucose‐responsive liposomes are self‐assembled on the aligned spun nanofibers. b) Constructed diabetic rat cranial defect model implanted with PCLA nanofibers. Subsequently, the responsive release of APY29 from PCLA in the high glucose environment is utilized to effectively regulate the immune microenvironment at the defect site and function with collagen to enhance the degree of vascularized bone regeneration and promote periosteum healing.

To achieve this goal, liposome surface modification with glucose‐responsive groups is employed, along with collagen self‐assembly and electrospinning to construct bionic microenvironment‐responsive fibers in a step‐by‐step manner (**Scheme** **1**a). More specifically, the FPBA on the surfaces of the spun‐film liposomes is expected to respond to a high‐glucose environment, leading to a change in the hydrophilicity of the liposome membrane. As a result, the exterior membrane swelling should increase, and the destruction of the lipid bilayer would result in the release of the encapsulated APY29. Subsequently, the released APY29 polarizes macrophages toward the M2 phenotype and induces the secretion of osteogenic and angiogenesis‐related factors. It is expected that the aligned electrospun scaffold will possess multiple functions, such as glucose response, osteoimmunomodulation, osteoinduction, and angiogenesis (**Scheme** 1b).

## 2 Results

### 2.1 Sample Fabrication and Characterization

**Figure 1:**
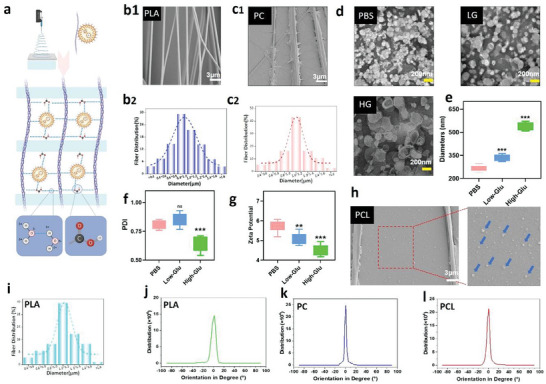
Manufacture and properties of the PLA, PLA/COLI, and PLA/COLI/Lip systems. a) Diagram of the manufacturing process used to obtain PLA/COLI/Lipo‐APY29 (PCLA). b1,b2,c1,c2) Scanning electron microscopy (SEM) images and particle size diameter distributions for the PLA and PLA/COLI systems. d,e) Transmission electron microscopy (TEM) and particle size diameters of the liposomes in the phosphate‐buffered saline (PBS), high‐glucose (HG), and low‐glucose (LG) groups. f) polydispersity index (PDI) values of the liposomes in the PBS, HG, and LG groups. g) Zeta potentials of the liposomes in the PBS, LG, and HG groups. h,i) SEM images and particle size diameter distribution of the PLA/COLI/Lip system. j,k,l) Distribution of the orientation degrees for the PLA, PLA/COLI, and PLA/COLI/Lip systems (n = 6 in each group, data are mean ± standard deviation [SD], *p < 0.05, **p < 0.01, ***p < 0.001, ns = no significance).

A poly(DL‐lactide)COOH solution on a parallel electrode receiving device was used to construct PLA by means of electrostatic spinning, as outlined in **Figure** **1**a. More specifically, the liposomes were mixed with DSPE‐PEG2K‐FPBA and APY29 to produce glucose‐responsive drug‐carrying nanospheres, which were then mixed with COLI following self‐assembly on the aligned spun nanofibers. The composite materials were obtained with a uniform distribution, robust plasticity and mechanical properties, and glucose‐responsive behavior.

Scanning electron microscopy (SEM) imaging confirmed a regular, directional, and smooth fiber distribution in the PLA group collected using a parallel electrode receiver (Figure 1b1). Following assembly of the PLA film with COLI, the thin‐film collagen was tightly combined by electrostatic spinning, and evenly laid on the spinning film to generate a micro/nanofiber hierarchical structure (Figure 1c1). Quantitative analysis showed that the average spinning diameters of PLA and PC are 1.04 ± 0.23 and 1.09 ± 0.20 µm, respectively, and their distributions were regular (Figure 1b2,c2).

Liposomes were dissolved in phosphate‐buffered saline (PBS), low glycemic (LG) medium, and high glycemic (HG) medium for 2 h to validate their glucose responsiveness. For the PBS group, transmission electron microscopy (TEM) imaging showed a typical phospholipid bilayer membrane structure in most liposomes. However, in the LG and HG groups, the bilayer structures of some liposomes were destroyed and their diameters increased, more significantly in the HG group (Figure 1d). To verify the changes in the core–shell structures of the liposomes under different glucose concentrations, dynamic light scattering was utilized to evaluate the size dispersion of liposomal microsol particles. The polydispersity index (PDI) was used to describe the degree of heterogeneity in particle size distribution, with relatively small PDI values implying a relatively uniform particle dispersion.^[^ ^19^ ^]^ It was found that upon increasing the glucose concentration in the solution, liposome particle size increased, while the surface charge and PDI decreased (Figure 1e–g). More specifically, the average diameter and PDI of the HG group microsol particles were determined to be 544.42 ± 24.90 nm and 0.64 ± 0.071, respectively; however, the PDI was found to be lower, which indicates that the particle size was uniformly dispersed.

The average diameters of the microsol particles in the LG and PBS groups were determined to be 341.52.00 ± 19.95 and 266.42 ± 21.89 nm, respectively, while their corresponding PDIs were 0.84 ± 0.065 and 0.81 ± 0.038. However, the particle distributions were less uniform than in the HG group. The data suggest that in the HG group, the liposomes without structural bilayer rupture were more stable and uniform in size. The reduced surface charge in the HG group can be due to the change in FPBA structure and the negative charge resulting from the combination of FPBA with glucose in the high‐glucose solution.^[^ ^20^ ^]^ As the immunomodulatory ability of the electrospun fibers mainly depends on their film‐like surface structure, SEM was used to investigate the assembly and architecture of the spun surface film. Compared with the PC group, the PLA/COLI/Lipo (PCL) group exhibited wrapped/spun collagen that was evenly laid over the surface. The liposomes on the PCL surface were also captured by the SEM view, indicating successful grafting (Figure 1h). To further verify the attachment of the liposomes to the spun surface, the liposomes were stained with a red cell membrane probe, and the PLA/COLI/Lipo‐APY29 (PCLA) structure was observed using confocal microscopy. The liposomes were found to follow the direction of spinning and adhered to the spinning surface (Figure S1a, Supporting Information). The average diameter of the liposome‐loaded electrospun PCL was 1.14 ± 0.28 µm, and many liposomes were uniformly distributed throughout the collagen structure (Figure 1i).

To characterize the orientation of the fiber scaffold, we set the overall orientation of the fiber membrane to 0°. Directional analysis is carried out through the OrientationJ plug‐in of ImageJ software.^[^ ^21^ ^]^ Bio‐oriented electrospun fiber scaffolds can also provide details regarding cell growth and expansion, both in vivo and in vitro. The orientation of the three sets of scaffold fibers in 0° were 154 058.67 ± 7567.71, 225 583.67 ± 11 523.99, and 253 744.00 ± 5855.88, indicating a highly consistent orientation in each group of scaffold fibers (Figure 1j–l).

**Figure 2:**
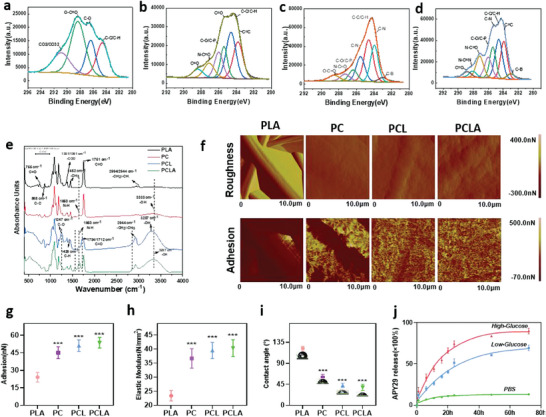
Characterization of the PLA, PC, PCL, and PLA/COLI/Lipo‐APY29 (PCLA) fibers. a,b,c,d) X‐ray photoelectron spectroscopy (XPS), e) fourier transform infrared (FTIR), and f) atomic force microscopy (AFM) spectra acquired for the PLA, PC, PCL, and PCLA fibers. g) Adhesion strengths. h) Elastic moduli. i) Water contact angles. j) High‐performance liquid chromatography (HPLC) traces showing APY29 release from the liposomes in phenylboronic acid (PBA) and high‐ and low‐glucose solutions (*n* = 6 per group, data are presented as the mean ± standard deviation [SD], ***p < 0.001).

X‐ray photoelectron spectroscopy (XPS) was then used to determine the alterations in chemistry on the fiber scaffold surface and to validate COLI and liposome construction. The results show that the PLA group elements are mainly C and O, whereas no signal was observed for Boron (B). In addition, as shown in **Figure** **2**a, the C 1s signal corresponds to polylactic acid. In terms of the PC group, the occurrences of C–P, C–N, N–C = O, and other components were confirmed in the C 1s spectrum, thereby confirming the successful introduction of COLI (Figure 2b). For the PCL group, C, O, N, and H were present, and a signal was also observed in the B 1s spectrum. These results indicate that the introduction of liposome/FPBA was successful, as further evidenced by the presence of a signal corresponding to the C─B bond in the C 1s spectrum (Figure 2c). Compared to the PCL group, the PCLA group contained a greater number of N─C═N bonds, thereby demonstrating the presence of APY29 (Figure 2d).

To further investigate the binding mode between the fiber scaffold, collagen, and liposomes, the chemical bonds of the four electrospun fibers (PLA, PC, PCL, and PCLA) (Table S1, Supporting Information) were identified using Fourier transform infrared (FTIR) spectroscopy (Figure 2e). The characteristic absorption peak observed at 1751 cm^−1^ for PLA corresponds to the C = O stretching vibration, which is typical of PLA.^[^ ^22^ ^]^ In contrast to the spectra observed for the electrostatically spun samples, a weak broad peak appeared at 3333 cm^−1^ for the composite product after COLI binding, which was mainly attributed to hydrogen bonding between the electrostatically spun fibers and collagen. After liposome binding, the –OH absorption peak at 3287 cm^−1^ shifted, confirming that the addition of drug molecules altered the hydrogen bonding forces present in the polymerization system. This was verified by the significant shift of the C–H absorption peak at 2944 cm^−1^ due to the weak hydrogen bonding between the drug‐related groups and liposomes/electrospun collagen. The disappearance of several characteristic peaks and the appearance of new ones confirm that the electrostatic spinning and subsequent binding to the collagen/drug‐loaded liposome complex was successful. Notably, the formation of this complex is mainly dependent on hydrogen bonding, particularly the strong hydrogen bonds between the collagen H–N groups and O–C moieties of the electrostatically spun fiber.

Graded micro/nanofibrous structures fabricated with collagen and electrospun fibers have the potential to direct the behavior of bone marrow mesenchymal stem cells (BMSCs), including adhesion, proliferation, and further differentiation, which are necessary for inducing cambium and ossification in vivo. To investigate this further, atomic force microscopy (AFM) was used to examine the changes in the surface roughness values (Ra, roughness) of the four groups of materials (Figure 2f); the surface roughness of the PLA, PC, PCL, and PCLA membranes was 65.17 ± 13.25, 6.81 ± 0.45, 12.87 ± 0.15, and 13.07 ± 0.25 nN, respectively (Figure S1b, Supporting Information). The obtained AFM phase diagrams showed the presence of circular, eminent liposomes on the membrane surface of PCLA, indicating stable consolidation of liposomes on the fiber surface. The adhesive abilities of the four groups of materials were measured; the adhesion strengths of the PC, PCL, and PCLA membranes were greater than those of the PLA membrane (24.66 ± 4.34, 43.82 ± 4.33, 49.52 ± 3.53, and 52.42 ± 3.74 nN for the PLA, PC, PCL, and PCLA membranes, respectively) (Figure 2g). This may be because the loading of COLI hydrogel enhances the tissue adhesion of the material compared to PLA electrospun.^[^ ^23^ ^]^ Furthermore, the enhanced adhesion observed in the presence of liposomes may be attributed to their H‐bonding interactions with COLI hydrogel.^[^ ^24^ ^]^ PCL and PCLA adhesion were slightly higher than those in the PC group, but there was no significant difference between the three groups. PCL and PCLA adhesion is slightly higher than that of PC, probably due to the enhanced liposome surface adhesion caused by PEG modification.^[^ ^25^ ^]^

As an important foundation for bone tissue engineering, the scaffold offers not just a growth platform for cells, but also guides the bone reconstruction process.^[^ ^26^ ^]^ Thus, the elastic moduli of the prepared specimens were evaluated, and the elastic moduli of PC (36.64 ± 1.26 N mm^−2^), PCL (39.49 ± 1.88 N mm^−2^), and PCLA (40.31 ± 0.81 N mm^−2^) were found to be higher than that of PLA (23.41 ± 0.81 N mm^−2^) (Figure 2h). These results were attributed to the self‐assembly of COLI, and the presence of hydrogen bonds between collagen and the electrospun surface, and between the liposomes and collagen. The improvement in mechanical properties was similarly reflected by higher maximum stress, maximum strain, maximum displacement, and maximum load for the PC, PCL, and PCLA specimens (Figure S2a–d, Supporting Information). The modulus of elasticity of the PCLA and PCL groups was slightly higher than that of the PC group, but it did not significantly differ between the PC, PCL, and PCLA groups. There is a weak hydrogen bond between liposomes and collagen, which may weakly influence the modulus of elasticity but not enough to significantly affect the change in the modulus of elasticity. Subsequently, the water contact angles of the PLA, PC, PCL, and PCLA fibers were determined to be 122.30 ± 4.49, 59.19 ± 7.27, 42.65 ± 4.62, and 38.59 ± 6.56°, respectively (Figure 2i). From these values, it is clear that the water contact angle decreased by ≈60° following the assembly of collagen, showing that the COLI nanofibers were clearly hydrophilic. Thus, the hydrophilicity of PCL and PCLA membranes was slightly higher than that of PC membranes, and the water contact angle of PCL and PCLA fibers declined significantly owing to the hydrophilic extension of phospholipids on the liposome surface into the aqueous phase to enhance hydrophilicity.^[^ ^27^ ^]^ The hydrophilicity of PCL and PCLA membranes is slightly higher than that of PC membranes, and the water contact angle of PCL and PCLA fibers decreases significantly. However, there is no significant difference in hydrophilicity between PCL and PCLA since phospholipid hydrophilia on the surface of liposomes extends to the aqueous phase to enhance hydrophilicity.^[^ ^27^ ^]^ At the same time, the liposomes of PEG coating will also be significantly more hydrophilic due to the modification of hydrophilic PEG.^[^ ^28^ ^]^

Diabetes leads to the formation of a high‐glucose environment in local areas of the bone. Based on this pathological feature, an in vitro simulation was used to detect the release of responsive drugs in a high‐glucose environment. More specifically, the drug (APY29) release rates of the three groups of liposomes (PBS, LG, and HG) at various time points (2, 4, 6, 12, 24, 48, and 72 h) were determined by soaking the electrospun membranes combined with drug‐loaded liposomes in culture media containing different glucose concentrations (high glucose: 25.0 mmol L^−1^; low glucose: 5.6 mmol L^−1^) (Figure 2j). In all three groups, it was observed that the degree of drug release from the liposomes gradually increased over time. However, in the high glucose group, the release rate of APY29 reached 86.19 ± 0.68% at 72 h, which was significantly higher than that of the LG group (65.09 ± 1.71%) and the PBS group (12.18 ± 0.38%). The rapid response of these reactive fiber scaffolds to a high‐glucose environment was therefore verified in vitro, providing a basis for further research. In addition, from the rheological study presented in Figure S1c(Supporting Information), the storage modulus (G′) was higher than the loss modulus (G″) in the Collagen I/liposome group, indicating that the assembled collagen formed a gel‐like state, which was retained after liposome addition. However, the fluidity of this group was stronger than that of the pure collagen group. Collagen is a triplet repeat sequence (XAA‐YAA‐GLY), where Xaa is usually proline (Pro), and Yaa is usually hydroxyproline (Hyp). The axial interaction of Xaa and Yaa is much stronger than the lateral interaction between them.^[^ ^29^ ^]^ Therefore, we speculate that due to the positive charge on the surface of liposomes modified with octadecylamine, the interaction with collagen is more lateral, affecting the relatively stable axial interaction between local collagen, such that stability is slightly affected and fluidity is slightly enhanced.

### 2.2 In Vitro Biocompatibility

**Figure 3:**
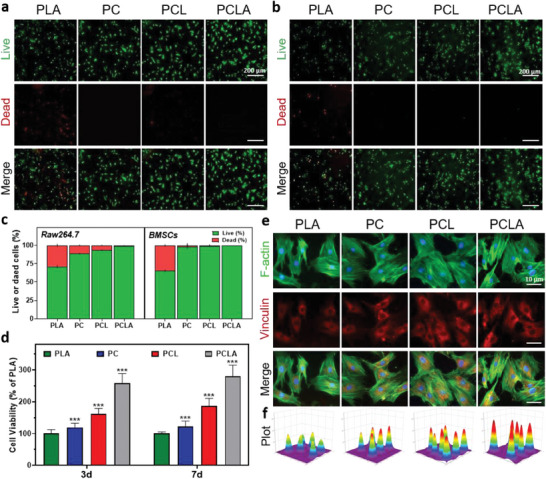
Cytocompatibilities of the spun PLA, PC, PCL, and PLA/COLI/Lipo‐APY29 (PCLA) fibers. a,b) Live/dead staining of RAW264.7 and bone marrow mesenchymal stem cells (BMSCs) after incubation in Dulbecco's Modified Eagle Medium (DMEM) for 72 h. c) Semi‐quantification of the live and dead cells (BMSCs and RAW264.7). d) Viability of the RAW264.7 cells after incubation in DMEM for 24 and 72 h. e,f) Vinculin staining of the BMSCs after incubation in DMEM for 72 h (n = 6 per group, data are provided as the mean ± standard deviation [SD], ***p < 0.001).

The in vitro biocompatibilities of the PLA, PC, PCL, and PCLA materials were subsequently evaluated by seeding mouse macrophages (RAW264.7 cells) and BMSCs on the surfaces of the spun materials. For this purpose, live/dead cell staining was initially performed. Compared with the surface of the bare PLA‐spun fiber, the numbers of dead cells in the PC, PCL, and PCLA groups were obviously reduced (**Figure** **3**a,b), as confirmed by quantitative analysis of the live/dead cells (Figure 3c). PLA will produce some acidic products during the degradation process, resulting in some toxicity to cells.^[^ ^30^ ^]^ Collagen is also a highly biocompatible material that provides an ideal environment for cell attachment and proliferation, making it an important candidate for tissue regeneration.^[^ ^31^ ^]^ Collagen nanofiber scaffolds can adhere, proliferate, and differentiate into osteoblasts as a matrix for osteoblast progenitor cells.^[^ ^32^ ^]^ Some studies with collagen nanofibers have shown adhesion rates in mesenchymal stem cell populations of >45% over a rapid reaction time of 30 min at room temperature (25 °C).^[^ ^33^ ^]^ Electrospun fibers and hydrogels are widely used in many biological and biomedical fields due to their unique structure and properties. On this basis, electrospun fibers and hydrogel composites are receiving increasing attention, aiming to capitalize on their individual advantages and compensate for their inherent defects.^[^ ^34^ ^]^ When a hydrogel is coated on a spinning surface, the hydrogel can act as a “dam” to intercept the release of the classic spinning‐encapsulated drug at the bottom of the hydrogel.^[^ ^35^ ^]^ In this experiment, type I collagen hydrogel coating on the surface of ordered spinning may also act as “dam” interception for the acidic environment caused by electrospinning degradation, so as to minimize the cell damage caused by polylactic acid degradation. To gain further insight into the proliferation of the RAW264.7 cells, the cell counting kit‐8 method was performed after coculturing the cells with the four fiber groups for 1, 3, 5, and 7 d (Figure 3d; Figure S3a and b, Supporting Information). On the first, third, fifth, and seventh days, we used PLA as a reference, set the PLA value to 100%, and then compared PC, PCL, and PCLA. The data show the relative value of the number of cells in the other groups relative to PLA at days 1, 3, 5, and 7. The RAW264.7 cells planted on the PC, PCL, and PCLA surfaces exhibited good viabilities, which may be due to the synergistic action of COLI and the liposomes to promote cell proliferation.^[^ ^16b^ ^]^

The morphologies and stretching characteristics of BMSCs on the different surfaces were subsequently examined using vinculin and F‐actin fluorescence, respectively. The BMSCs cultured on the spun PLA for 3 d showed short filamentous feet, while those cultured on the surfaces of the PC, PCL, and PCLA groups showed improved cell adhesion properties, a polygonal shape, and longer filamentous feet (Figure 3e). Similarly, the fluorescence intensity of vinculin in the PC, PCL, and PCLA groups was higher than that in the PLA group, which indicated that the biocompatibility of the PLA‐spun fibers—which initially exhibited a poor surface adhesive ability—was significantly improved following the incorporation of COLI and liposomes; this material, therefore, appears suitable for use in osteogenesis. The vinculin fluorescence intensities of the four groups of materials were also analyzed semi‐quantitatively; the fluorescence intensity of vinculin in the PCL and PCLA groups was obviously higher than that in the PLA group (Figure 3f). The blood compatibility of biomaterials in contact with blood is important for their successful implementation in vivo. The materials should not have adverse reactions with any blood components, whether activating or destroying them.^[^ ^36^ ^]^ The PLA, PC, PCL, and PCLA specimens were all negative for such reactions (Figure S4a,b, Supporting Information). Overall, the aforementioned results show that the electrospun surfaces bearing COLI and liposomes promote macrophages and BMSC growth with no apparent cytotoxic effects.

### 2.3 Effects of the PCLA‐Based Regulation of The Immune Response Under Simulated DM Conditions

**Figure 4:**
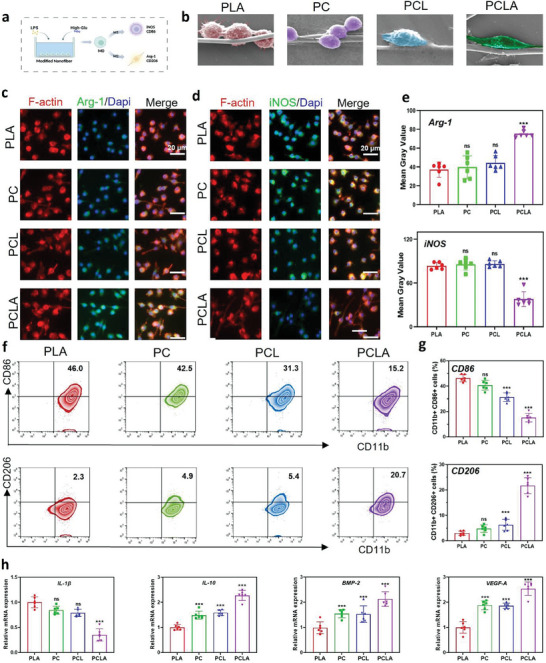
Regulation of the macrophage‐polarization phenotype in vitro. a) Illustration of PLA, PC, PCL, and PLA/COLI/Lipo‐APY29 (PCLA) modulating macrophage polarization. b) Scanning electron microscopy (SEM) images of Raw264.7 seeded on the PLA, PC, PCL, and PCLA surfaces. c,d) Immunofluorescent staining images of the cells (green: cytoskeleton; M2 marker Arg‐1; red: M1 marker iNOS; blue: nuclei) (scale bar = 20 µm). e) Semi‐quantitative results of Arg‐1 and iNOS. f,g) Percentages of CD11b+CD86+ and CD11b+CD206+ RAW264.7 cells cultured on the different material groups, as determined by flow cytometry. h) Real‐time quantitative reverse transcription polymerase chain reaction (RT‒qPCR) results of (*IL*‐1β*, IL‐10, BMP‐2, VEGF‐A*) gene expression in RAW264.7 cells cultured in osteogenic medium in a diabetes mellitus (DM)‐mimicking environment (n = 6 per group, data are provided as the mean ± standard deviation [SD], *p < 0.05, ***p < 0.001, ns = no significance).

Osteoimmune cells are indispensable for osteogenesis and angiogenesis, particularly in the early immune environment.^[^ ^37^ ^]^ To study the ability of PCLA to reprogram macrophages in the diabetic inflammatory microenvironment, the environment was initially simulated using a high‐glucose medium and lipopolysaccharides (LPS), and subsequently, the phenotypic changes of the medium macrophages seeded on the PCLA surface were examined (**Figure** **4**a).^[^ ^38^ ^]^ In the PLA, PC, and PCL groups, the macrophages mainly adopted a pancake‐like morphology, whereas, in the PCLA group, they were elongated (Figure 4b). Additionally, the fluorescence intensities of the M2 polarization‐related proteins markedly increased in the elongated cells on the PCLA surface, illustrating that the macrophages were polarized from the pro‐inflammatory M1 status to the anti‐inflammatory M2 status. As shown in Figure 4c,d, M2 macrophages (Arg‐1+, green) were significantly increased in the PCLA group than in the other three groups. In contrast, M1 macrophages (iNOS+, green) were predominant in the PLA group. It is worth mentioning that compared to the PLA group, Arg‐1 expression in the PC and PCL groups was slightly upregulated, whereas iNOS was slightly downregulated in semi‐quantitative analysis (Figure 4e).

Flow cytometry was used to further explore the M1–M2 phenotype switch at the cellular level (Figure 4f,g). The CD11b+/CD206 ratio of the APY29‐loaded stents was significantly higher than that of the APY29‐free stents, while the corresponding ratios in the PC, PCL, and PLA groups also increased significantly after the addition of COLI,. Similarly, the expression of CD11b+/CD86 in the PCLA group was significantly lower than that in the PLA, PC, and PCL groups. These significant differences imply that PCLA is robustly capable of manipulating macrophage behavior and strongly influences the immune environment. Next, we detected a number of inflammatory and anti‐inflammatory factors; the performance of pro‐inflammatory factors, such as interleukin‐1β (IL‐1β), was higher in the PLA group, while the expression of anti‐inflammatory factor IL‐10 was higher in the PCLA group. Thus, it seems that PCLA significantly inhibited the secretion of pro‐inflammatory cytokines and promoted the expression of anti‐inflammatory cytokines. Additionally, the key role of osteogenesis‐related genes in regulating osteogenesis and angiogenesis was well elucidated; the expression of osteogenic and angiogenic‐related markers (BMP‐2 and VEGF‐A) was significantly enhanced on PCLA (Figure 4h). In summary, PCLA down‐regulated inflammatory responses, up‐regulated anti‐inflammatory factors, activated macrophage conversion to the M2 phenotype, and released large amounts of osteogenic/angiogenic mediators; thus, it can be considered as a potent and beneficial bone immunomodulatory material.

### 2.4 Effects of The Electrospun Scaffold Surface On Osteogenesis and Angiogenesis

Properties of the implant surface can control the behavior of the BMSCs and, thus, the extent of new bone formation,^[^ ^39^ ^]^ with angiogenesis being a prerequisite for achieving bone formation.^[^ ^40^ ^]^ Considering this, in an effort to test the impact of electrospun scaffolds on osteogenic activity, BMSCs were seeded onto the membrane surface to assess the direct osteogenic and angiogenic capabilities of the electrospun material. As shown in Figure S5a—c (Supporting Information), alkaline phosphatase (ALP) staining and alizarin red (ARS) mineralization of ECM indicated that PC, PCL, and PCLA promote the osteogenic differentiation of BMSCs. Notably, the osteogenic effect was significantly higher in the PC and PCL groups than in the PLA group, which may be due to the osteogenic effect of COLI.^[^ ^26^ ^]^ Simultaneously, endothelial cell‐mediated angiogenesis is also essential to achieving bone formation, remodeling, and osseointegration.^[^ ^41^ ^]^ Hence, owing to the dependence of successful bone regeneration on vascularization, human umbilical vein endothelial cells (HUVECs) were cultured on the four spun scaffold materials to evaluate their angiogenic activities. The angiogenic capacity of each specimen‐stimulated HUVECs were then assessed using an in vitro angiogenesis test set containing ECM gel. However, migration experiments showed no significant differences between the groups of materials in regard to facilitating cell migration. Similarly, crystal violet staining and tube formation experiments showed that the angiogenic abilities of PC, PCL, and PCLA were not significantly different from those of the PLA group, indicating that PCLA does not have the ability to directly promote the formation of blood vessels (Figure S6a—c, Supporting Information).

### 2.5 Coupling of osteoimmunomodulation with osteogenesis and angiogenesis

**Figure 5:**
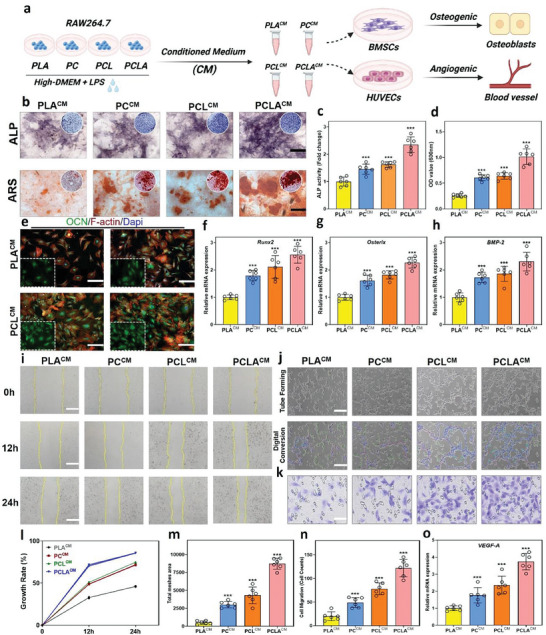
Immunomodulation‐enhanced osteogenic and angiogenic differentiation in vitro. a). Description of the experimental setup; b) alkaline phosphatase (ALP) and alkaline phosphatase (ARS) staining of bone marrow mesenchymal stem cells (BMSCs) in cultures for 7 and 21 d (scale bar = 10 mm). c) Quantitative evaluation of ALP activity. d) Quantification of ARS at optical density value (490 nm). e) Immunofluorescent staining images of the BMSCs (green: osteocalcin (OCN); red: cytoskeleton; blue: nuclei) (scale bar = 10 µm). f–h) Real‐time quantitative reverse transcription polymerase chain reaction (RT‒qPCR) results for osteogenesis‐related gene expression (Runx2, Osterix, and BMP‐2) in BMSCs cultured in CM. i) Representative images of human umbilical vein endothelial cell (HUVEC) migration at 0, 12, and 24 h in an in vitro scratch wound healing assay and in vitro tube formation (scale bar = 200 µm). j,k) Tube forming experiment (scale bar = 100 µm) and migration assays (scale bar = 200 µm) were used to assess angiogenesis in different groups. l) Quantitative migratory area analysis. m,n) Quantitative tube formation analysis, showing total mesh area and the number of migrating cells/fields. o) Quantitative VEGF expression analysis (6 per group, the data are mean ± standard deviation [SD], ***p < 0.001).

Osteommunomodulation is emphasized by the manipulation of immune cells by biomaterials to generate a beneficial bone immune environment, thereby, regulating the formation of new bone.^[^ ^42^ ^]^ After an early immune response by macrophages, HUVEC and BMSC cells are recruited to the implant surface, while the function of HUVECs and BMSCs is mostly influenced by the macrophage‐regulated immune microenvironment.^[^ ^43^ ^]^ To investigate the coupling of osteoimmunomodulation with osteogenesis and angiogenesis, RAW264.7 cells pretreated with PLA, PC, PCL, and PCLA were stimulated with LPS and collected in a conditioned medium (CM) (**Figure** **5**a). The osteoimmunomodulatory function of PCLA was then evaluated by collecting the macrophage CM for culturing BMSCs and HUVECs. ALP staining showed that ALP activity in the PC, PCL, and PCLA groups was significantly higher than that of the PLA group (Figure 5b,c). In addition, on day 21, ARS staining of the calcium‐binding proteins in the mineralized matrix revealed that the number and size of mineralized nodes in the PCLA group were significantly larger than those of other groups (Figure 5b,d). Osteocalcin (OCN) staining also confirmed that the relative expression of OCN was significantly higher in the PCLA group than that in the PLA, PC, and PCL groups and that osteogenic differentiation was also significantly enhanced in the PCLA group (Figure 5e). The expression levels of other genes (Runx2, Osterix, and BMP‐2) related to osteogenesis were also examined (Figure 5f–h), and their expression was significantly higher in PCLA than in the other groups. These results reveal that PCLA had a greater capacity for osteogenic differentiation compared with other groups, which may be related to the immunomodulatory properties of APY29 that prompted macrophages to secrete the osteogenic growth factor BMP‐2.^[^ ^7^, ^44^ ^]^

The behavior and function of ECs are influenced by the immune microenvironment generated by the MΦs regulated by the material.^[^ ^7^, ^45^ ^]^ Angiogenesis is an indispensable biological process before osteogenesis in bone repair and is considered a prerequisite for bone regeneration and integration.^[^ ^46^ ^]^ The results of EC migration under CM stimulation are shown in Figure 5i, and it is clear that the exposed gap can gradually close due to the directed migration of cells. Compared with the control group, CM‐cultured HUVECs in the PCLA group showed more significant migration ability. Semi‐quantitative analysis showed that the wound closure area in the PCLA group was significantly smaller than that in the other three groups after 12 and 24 h (Figure 5l), and the most significant EC migration ability was shown in the >80% cell mobility and the near‐closed gap. As shown in the optical images of the capillary network in Figure 5j, there was no evidence of capillary network formation in the PLA group. The CM of PCLA showed significantly enhanced formation of capillary‐like structures (e.g., tubes, nodes, and branches) compared with the other specimens (Figures 5m and n). In addition, the migration of HUVECs was analyzed by crystal violet staining, and it was found that more PCLA^CM^ cells switched to the other side of the Transwell membrane. In fact, few metastatic cells were observed in the other groups (Figure 5k). Subsequently, VEGF secretion was detected 72 h after culture as an indicator of HUVEC activity (Figure 5o).^[^ ^47^ ^]^ The transcriptional expression of VEGF‐A is superior in PCLA^CM^ in promoting VEGF‐A secretion by Raw264.7, and the expression of angiogenesis‐related genes is the highest. This may be attributed to the contribution of PCLA to the regulation of the immune microenvironment; therefore, PCLA is associated with regulating the immune microenvironment related to VEGF‐A secretion by macrophages.^[^ ^44^, ^48^ ^]^ Taken together, these results suggest that a favorable bone microenvironment leads to robust osteogenic and angiogenic biological activity.

### 2.6 RNA‐Seq Verification of Osteoimmunomodulation of PCLA

**Figure 6:**
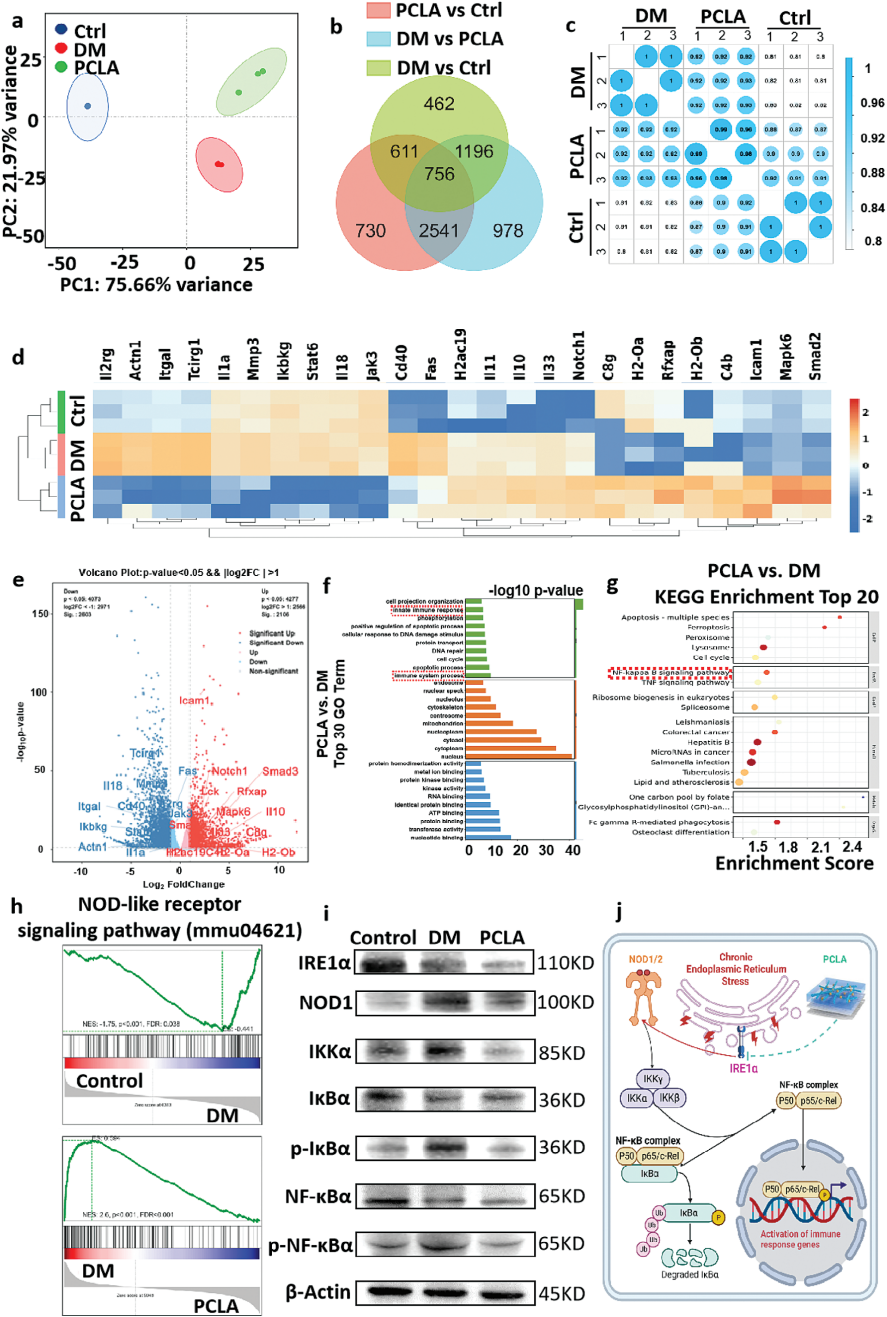
Cells from the control, diabetes mellitus (DM), and PLA/COLI/Lipo‐APY29 (PCLA) groups were analyzed through transcriptome sequencing. a) PCA of samples collected from the three groups (control, DM, and PCLA groups). b,c) Differential gene analysis and intersample correlation tests, and d) Gene ontology (GO) enrichment analysis circle diagram of the differential groups (control, DM, and PCLA). e) Expression heatmap of the differentially expressed genes (brown: higher expression; blue: lower expression). f,g) GO and KEGG enrichment analyses (DM versus PCLA). h,i) Heatmaps and volcano plots of the inflammation‐related genes (control versus DM). j,k) Correlation analysis of the NOD‐like signaling pathway (control versus DM, and DM versus PCLA). l) Western blot for detection of the IREα/NOD‐like/NF‐κB signaling pathway‐activated proteins. j）Schematic representation of the mechanism of PCLA by blocking the IREα/NOD‐like/NF‐κB signaling pathway.

To further explore the mechanisms by which PCLA regulates immunity, RNA sequencing was conducted to demonstrate that PCLA has a satisfactory ability to reduce the expression of inflammatory response‐related genes in macrophages growing in a DM environment, which prompted us to further reveal the potential mechanisms and related biological events in macrophages treated with PCLA. Principal component analysis and intersample correlation tests showed that each group of samples demonstrated good reproducibility (**Figure** **6**a,b). Compared to the PLA group, the DM and PCLA groups exhibited 1367 and 1952 differentially expressed genes, respectively, and 3297 genes were observed with significant differences between the DM and PCLA groups (Figure 6c). Enriched Gene Ontology (GO) analysis showed that molecular function and a range of cellular components played significant roles in determining the differences between the PCLA and DM groups, including protein binding, nucleotide binding, transferase activity, centrosomes, mitochondria, nucleoplasm, and the cytosol (Figure S7, Supporting Information). Thousands of genes with marked variations were identified between the control, DM, and PCLA groups, and compared to the control group, many inflammatory pathways were activated in the DM group (Figure 6d; Figure S8, Supporting Information). Volcano map and DESeq2 variance analyses showed that compared with the DM group, the PCLA group possessed 2071 upregulated genes and 2567 downregulated genes. Among the differentially expressed genes in the DM group, the majority of immunity‐ and inflammation‐related genes were upregulated compared to those in the PLA group; in contrast, they were downregulated in the PCLA group. As shown in Figure 6e, the volcano maps clearly indicated the locations of the pro‐ and anti‐inflammatory factors in all significantly and differentially expressed genes.

The GO analysis showed that for PCLA‐induced changes, the main concerns were immune and inflammatory reactions. In particular, PCLA is mainly engaged in the negative modulation of the inflammatory reaction and production of molecular mediators associated with the inflammatory response (Figure 6f). Analysis of the enrichment of the Kyoto Encyclopedia of Genes and Genomes (KEGG) pathway further suggested that PCLA interacts strongly with the NF‐kappa B signaling pathway, the TNF family, and other inflammation‐related pathways (Figure 6g). The inflammation‐related genes that were upregulated and downregulated in the PCLA group were then examined further. Among the related inflammation genes, when comparing the PCLA and DM groups, the reversal of the pro‐inflammatory genes and elevation of the anti‐inflammatory genes was significant. This suggests that PCLA effectively alleviates inflammation by influencing multiple inflammation‐related pathway genes (Figure S9, Supporting Information). In addition, Gene Set Enrichment Analysis demonstrated that a high‐glucose environment enhanced the expression of inflammatory factors via NOD‐like signal transduction pathways in the DM group, while the PCLA group was negatively correlated with the NOD‐like signaling pathways (Figure 6h). Above all, the immunoregulatory function of PCLA may originate from multi‐pathway regulation because these differentially expressed genes are involved in the NF‐κB and NOD‐like signaling pathways that affect the inflammatory response. It was therefore speculated that PCLA has the potential for regulating bone homeostasis by modulating the IREα/NOD‐like/NF‐κBsignaling pathway.^[^ ^49^ ^]^ To test this hypothesis, the protein expression of these pathways was characterized by western blotting, and it was confirmed that PCLA effectively inhibited the phosphorylation of key proteins in these pathways (Figure 6i). The schematic diagram in Figure 6j exhibits the way in which PCLA blocks the activation of immune response genes by inhibiting the IREα/NOD‐like/NFκB signaling pathway.

### 2.7 In Vivo Phenotypic Transformation Of Macrophages

**Figure 7:**
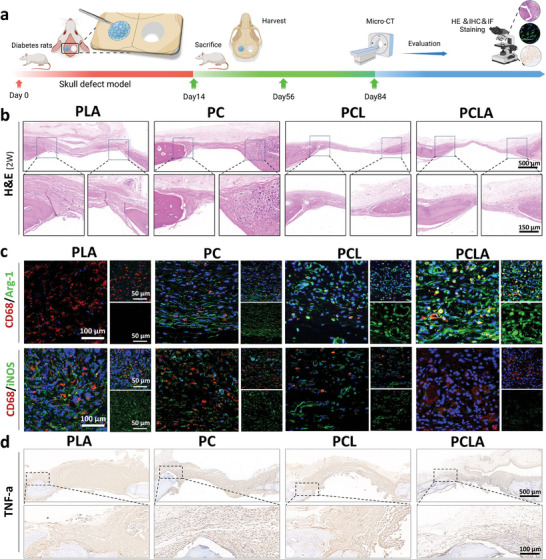
In vivo modulation of the macrophage polarization phenotype. a) Schematic diagram of the modeling and animal experiment procedures. b) H&E stained image of the tissue in the area of the bone defect (scale bar = 100 µm). c) Immunofluorescence of the RAW264.7 cells showing green (M1 marker: iNOS; M2 marker: Arg‐1), red (CD68, a monoclonal antibody specifically targeting mouse macrophages), and blue (4′,6′‐diamidino‐2 ‐phenylindole, DAPI) components. d) Immunohistochemistry of the TNFα inflammatory factor.

Following examination of the macrophage immune regulation and osteogenesis regulation in vitro, osseointegration was further studied in a diabetic animal model. According to animal operating rules, Streptozotocin injection was used to establish an animal model of diabetes (**Figure** **7**a).^[^ ^50^ ^]^ In this study, the outcomes of the in vitro research prove that PCLA surface exhibits improved cell compatibility, accelerated cell growth, effective regulation of the macrophage phenotypes, and a strong bone immunomodulatory activity, ultimately enhancing osteogenesis.

In vivo experiments to study osteogenesis and immunomodulatory activity were conducted for all four groups (PLA, PC, PCL, and PCLA). The rat skull defect model was constructed according to a standard surgical protocol (Figure S10a,b, Supporting Information). The immunomodulatory effect of the macrophage phenotype mainly manifested in the early and middle stages. Therefore, we initially collected animal bone tissues for testing in the early stages of the model. Two weeks after implantation, the skull implanted with the spinning film was stained with hematoxylin and immunofluorescence (H&E) stain for histological study. The thick fibrous layer around the material and massive infiltration of inflammatory cells were the main factors contributing to the failure of the bone defect. The fibrous layer thickness and the numbers of inflammatory cells were detected by H&E staining. As presented in Figure 7b, the H&E images revealed that, compared with the PLA group, the PC, PCL, and PCLA groups exhibited reduced inflammatory reactions and thinner fibrous layers, wherein the PCLA group exhibited the lowest inflammatory response and the thinnest fiber layer.

Immunostaining was performed to further evaluate the phenotypic transformation of the macrophages during spinning. Specifically, the phenotypic transformation from M1 to M2 was mediated by labeling iNOS‐ and Arg‐1‐positive cells (Figure 7c), as confirmed by immunofluorescence images of the PC, PCL, and PCLA groups. The amount of Arg‐1‐positive cells (i.e., M2 macrophages) in the three groups was noticeably larger than that in the PLA group. In comparison, the PLA group had a higher number of iNOS‐positive cells (M1 macrophages) (Figure 7d). This transformation and regulation of the macrophage phenotype create a beneficial immune microenvironment that enhances osteogenic differentiation and bone remodeling surrounding the implant. Overall, these results indicate that PCLA can regulate the macrophage phenotype in vivo, increase the degree of transformation from the M1 to the M2 phenotype, and inhibit the overactivation of pro‐inflammatory macrophages. The PCLA group showed stronger immunoregulatory activity in vivo than the other groups, indicating its ability to reverse over‐validation in diabetic rats, and its potential to enhance osseointegration of the interface around the material.

### 2.8 Evaluation Of In Vivo Performance

**Figure 8:**
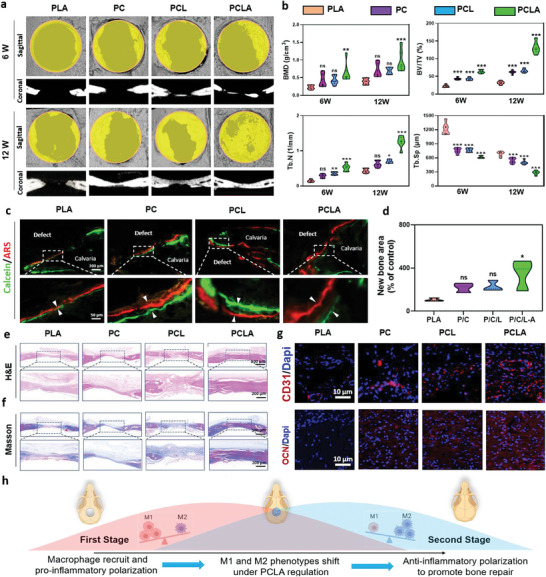
In vivo regulation of bone remodeling by PLA/COLI/Lipo‐APY29 (PCLA). a) Micro‐CT scanning and reconstructed 3D images of cranial bone defects containing different scaffolds. b) Quantitative evaluation of bone generation in the defect region according to the BMD, BV/TV, Tb.N, and Tb.Sp (n = 6 per group, data are provided as the mean ± standard deviation [SD], *p < 0.05, **p < 0.01, ***p < 0.001, ns = no significance). c) Calcein and Alizarin red fluorescence of the cranial bone tissue. d) Quantification of the new bone mass. e,f) H&E and Masson staining images of the cranial defect tissue (scale bar = 100 µm). g) Immunofluorescence staining of the osteogenesis‐related markers in the peri‐implant tissue (red: CD31 and osteocalcin (OCN); blue: DAPI). h) Schematic diagram of macrophage switches in diabetes mellitus (DM) microenvironment of the calvarial periosteal defect after PCLA implantation.

To determine whether PCLA promotes bone fusion in diabetic rats, different groups of materials were implanted into the skull defect surfaces according to the aforementioned grouping arrangement. At 8 and 12 weeks after implantation, new bone formation in the skull defects was evaluated. 3D reconstruction of micro‐CT images was performed along with histological analysis after obtaining the rat skull. The 3D image displayed the presence of a large amount of newly formed bone tissue in the PCLA group after 6, 8, and 12 weeks, while very little newly formed bone tissue was found in the composite implanted PLA group (**Figure** **8**a; Figure S11, Supporting Information). This was confirmed by quantitative analysis, in which the PCLA group was found to have the highest bone mineral density (BMD), bone volume/tissue volume (BV/TV), and trabecular number (Tb.N) but the lowest trabecular separation (Tb.Sp) (Figure 8b). Under the same volume of interest, the new trabecular bone exhibited superior structural characteristics. As expected, PCLA exhibited the strongest bone‐forming ability, which is likely due to the synergistic effects of APY29 and COLI. In contrast, collagen alone may fail to offer the best immune regulatory microenvironment for the regeneration of bone.

The newly formed bone was sequentially labeled with calcitonin (green) and Alizarin red (red), and the results were similar to those described above (Figure 8c). Specifically, large areas of new bone mineralization were observed in the PCLA (18.80%), PCL (11.10%), PC (8.22%), and PLA (3.74%) groups (Figure 8d). To further evaluate the impact of various treatments on rat cranial defects, histological staining was performed on the harvested samples 12 weeks post‐surgery. The H&E and Masson stained images of the cranial defects after the different treatments showed a greater degree of new bone formation in the PCLA, PCLA, and PC groups than in the PLA group; the largest bone value was observed in the PCLA group after 12 weeks, indicating a superior extent of osteogenesis (Figure 8e). In addition, Masson staining showed the appearance of blue‐stained new bone tissue and red‐stained cancellous bone in the PCLA group, indicating an increase in calcification, maturation, and remodeling of new bone tissue (Figure 8f). Immunofluorescence showed that OCN and CD31 were highly expressed in cells implanted with PCLA, PCL, and PC, wherein the PCLA group showed the highest levels of OCN and CD31 expression (Figure 8g). Overall, PCLA led to the enhanced promotion of osteogenesis and angiogenesis in vivo compared to the other materials, suggesting its potential for postoperative therapy of cranial defects in rats.

In addition, in vivo toxicity of the implanted spinning material against the heart, liver, spleen, lungs, and kidneys was assessed; notably, no obvious tissue toxicity was detected (Figure S12, Supporting Information). Subsequently, the routine blood and biochemical indices of rats implanted with PCLA and those without material implantation were compared, with no apparent changes observed among the two groups (Figure S13, Supporting Information). These results clearly demonstrate that bone immunomodulation and regeneration can be carried out simultaneously and may promote one another. A biomaterial that enhances both bone induction and immunomodulation was obtained using the aligned spun fiber as a carrier in combination with COLI and with PCLA‐loaded lipids, wherein PCLA was modified with a glucose response group. Such dual‐effect biomaterials not only induce phenotype switching from M1 to M2 in the early stage but also induce direct osteogenesis, cooperatively creating an in vivo microenvironment conducive to bone regeneration (Figure 8h). To date, glucose‐responsive immunomodulatory liposomes have not been combined with osteogenic collagen and their synergistic effect on osteogenic differentiation has not been clarified.

## 3 Discussion

Chronic tissue inflammation has emerged as a key feature of diabetes. The recruitment, accumulation, and activation of pro‐inflammatory macrophages in metabolic tissues is the ultimate driver of this chronic low‐grade inflammation. Therefore, the macrophage is considered the major effector cell type.^[^ ^51^ ^]^ Concomitant glycemic fluctuations often occur in patients with poor glycaemic control and are considered an independent risk factor for the development of chronic diabetic complications.^[^ ^52^ ^]^ Reducing glucose fluctuations should be considered an important goal in controlling diabetes and tissue regeneration.^[^ ^53^ ^]^ Dysfunction of metabolism and dysregulation of inflammation caused by diabetes are the main causes of delayed tissue healing^[^ ^54^ ^]^—the impairment of diabetic wound healing is also multifactorial.

The inability of normal tissue macrophages to transition from the M1 to M2 phenotype around the third day after injury is an important distinction between normal and diabetic wound healing. In patients with diabetes, several factors that contribute to the sustained stimulation of M1 macrophages have been identified; chief among them, high blood glucose, which leads to a persistent inflammatory state that promotes the M1 macrophage phenotype.^[^ ^55^ ^]^ General biomaterials in the treatment of diabetic bone defects pay the most attention to bone and vascular regeneration and alleviation of the hyperglycemic environment through glucose oxidase and glucose interaction.^[^ ^56^ ^]^ However, for complex chronic diabetic environments, blood glucose fluctuations cannot be dynamically responded to by adjusting the amount of drug released.

Lipid nanoparticle‐based drug delivery systems have profound implications for diabetic‐based tissue damage repair.^[^ ^55^, ^57^ ^]^ Liposomes are nanocarriers that protect the active ingredients they carry from degradation.^[^ ^58^ ^]^ They are currently considered the most successful drug carrier systems.^[^ ^59^ ^]^ The main method of fine‐tuning these nanocarriers is surface modification. The most common practice is to add PEG molecules to liposomes. Polyethylene glycolated liposomes are generally considered safe, effective, and extremely useful and are currently used in the treatment of various diseases. PEG liposomes are being studied in greater depth, which has led to the discovery of the benefits of ligand attachment.^[^ ^60^ ^]^

Stimulus‐response systems have emerged as a promising method for site‐selective delivery and release of payloads, with carrier systems derived from various materials, including lipids, inorganic nanoparticles, and polymers with stimulus‐sensitive properties to complete trigger release. Triggered release is usually based on the principle that local defects within the bilayer membrane lead to membrane instability to achieve the release of liposome‐embedded drugs. Depending on drug properties, the drug can be incorporated into an aqueous nucleus or a bilayer membrane. Ligands can be introduced for presentation on the surface to manage specific binding, while drug release can be dynamically controlled by designing sensitivity to specific stimuli.^[^ ^61^ ^]^

APY29 is an IRE1α inhibitor with the ability to enhance M2 polarization, and through reasonable design, the modified positively charged liposomes on the surface can be delivered with the glucose‐sensitive element phenylboronic acid and released in a glucose‐responsive manner. In this study, in order to dynamically and responsively regulate the immune microenvironment using a glucose‐responsive drug release strategy, a glucose response group—FPBA—was first constructed. Specific glucose response mechanisms include FPBA, a type of Lewis acid that can combine with 1,2 or 1,3 hydroxyl groups (glucose) to form a dynamic borate ester structure, which changes from being hydrophobic to hydrophilic, increasing its swelling degree. On the surface of modified liposomes, the double‐layer structure is destroyed, pores are formed, and loaded drugs are released under a high‐glucose environment. Therefore, FPBA can be used as a functional group to realize the glucose response.

Regulation of the local immune microenvironment and in situ bone formation by mesenchymal stem cells is essential in diabetic periosteal defects. Therefore, liposomes can be loaded with APY29 and self‐assembled with COLI on the aligned spinning surface. In terms of specific functions, APY29 is a specific IRE1α allosteric modulator that inhibits the autonomic phosphorylation of IRE1α and enhances M2 polarization. Aligned spinning easily guides the axial mineralization of BMSCs, while COLI has osteogenic and self‐assembly abilities. Therefore, glucose‐responsive liposomes can effectively repair diabetic bone defects, and aligned spinning can be used as a platform for cell osteoblastic differentiation and proliferation. Our follow‐up experimental results also confirmed that the rate of drug release for different glucose concentrations is also different, and the higher the glucose concentration, The faster the encapsulated drug is released.

In the present study, we used lipopolysaccharide and high‐glucose medium to simulate the diabetic inflammatory microenvironment.^[^ ^62^ ^]^ A certain degree of inflammatory response is important for bone repair after implantation, but excessive inflammation leads to a decline in local bone regeneration and impaired osseointegration.^[^ ^63^ ^]^ Our findings suggest that LPS‐induced inflammation causes abnormal inflammatory cytosis around the implant and dysplasia of scar tissue that impairs osteogenic activity. In the experimental group, PCLA was shown to mediate macrophage polarization to the M2 phenotype in both in vivo and in vitro inflammatory states, preventing activated macrophages from producing proinflammatory mediators and promoting osteogenic and vasogenic activities as well as osseointegration.

Overexpression of systemic or local pro‐inflammatory factors is the key cause of implant failure.^[^ ^46^ ^]^ Cytokines such as IL‐1 β and IL‐6 disrupt the homeostasis between osteoblast bone formation and osteoclast bone resorption.^[^ ^64^ ^]^ IL‐10 plays an important role in the control and resolution of inflammation, and some studies have shown that IL‐10 can promote mitophagy to eliminate dysfunctional mitochondria characterized by low membrane potential and high levels of reactive oxygen species.^[^ ^65^ ^]^ Our results indicate that LPS increased proinflammatory cytokine production in M1 macrophages and decreased the expression of the IL‐10 anti‐inflammatory factor. However, in the experimental group, PCLA could reduce the expression of the LPS‐induced M1 phenotype and increase the expression of the M2 phenotype, thus demonstrating that PCLA could convert M1 macrophages into the M2 phenotype, reducing proinflammatory factor production and promoting bone regeneration.

To further explore the mechanisms by which PCLA regulates immunity, transcriptome sequencing was performed. The results showed that PCLA has strong interactions with NF‐κB signaling, TNF‐α, as well as other inflammation‐related pathways to effectively relieve inflammation by affecting multiple inflammation‐related pathway genes. Inhibition of NF‐κB signaling transformed M0‐type macrophages into M2‐type macrophages with an anti‐inflammatory function.^[^ ^66^ ^]^ There is also evidence that NF‐κB is an important regulator of bone remodeling, and blocking NF‐κB activation suppresses inflammatory bone destruction.^[^ ^67^ ^]^ Gene set enrichment analysis indicated that the high glucose environment enhanced the expression of inflammatory factors through the NOD‐like signal transduction pathway in the DM group, whereas the PCLA group was negatively associated with NOD‐like signaling. These results suggest that PCLA regulates bone homeostasis by regulating the IREα/NOD‐like/NF‐κB signaling pathway. To test this hypothesis, the protein expression of these pathways was verified by western blotting, confirming that PCLA effectively inhibited the phosphorylation of key proteins in these pathways. In conclusion, our results suggest that PCLA regulates macrophage phenotype by inhibiting NF‐κB signaling and secretion of proinflammatory cytokines, effectively promoting bone formation under inflammatory conditions.

## 4 Conclusion

The pathological diabetic microenvironment, which consists of glucose fluctuations and chronic inflammation, leads to the delayed healing of periosteal defects. It is therefore necessary to develop new materials that are conducive to bone integration and suitable for use in the complex microenvironment of diabetes. This study reports a novel glucose‐responsive PCLA composite that modulates the local immune microenvironment and promotes bone integration at the periosteum‐implant interface. It was found that PCLA remodels the pathologic diabetic microenvironment into a regenerative microenvironment by blocking the IREα/NOD‐like/NF‐κB signaling pathway, while also reprograming macrophage shift from the M1 to M2 phenotype and enhancing osteogenic differentiation and angiogenesis. Collectively, our findings suggest that PCLA regulates the immune microenvironment and enhances the osseointegration of periosteal defects in patients with diabetes. Moreover, this study provides an effective strategy for designing functionalized biomaterials for bone regeneration therapy in diabetic patients.

## Conflict of Interest

The authors declare no conflict of interest.

## Supporting information

Supporting Information

## Data Availability

Research data are not shared.
