# Supplementary material for: Spatiotemporal Immunomodulation and Biphasic Osteo‐Vascular Aligned Electrospun Membrane for Diabetic Periosteum Regeneration
Source: Adv Sci (Weinh). 2023 Nov 16;10(36):2302874. doi: 10.1002/advs.202302874 (PMC10754081; doi:10.1002/advs.202302874)
Supplement: Supplementary file 1 — Supporting Information [file ADVS-10-2302874-s001.pdf]

ADVANCED SCIENCE
Open Access

Supporting Information

for *Adv. Sci.*, DOI 10.1002/advs.202302874

Spatiotemporal Immunomodulation and Biphasic Osteo-Vascular Aligned Electrospun Membrane for Diabetic Periosteum Regeneration

Yusen Qiao, Lei Yu, Peng Yang, Miao Chen, Haifu Sun, Lingjie Wang, Bangzhao Wu, Chun-do Oh, Huilin Yang, Jiaxiang Bai* and Dechun Geng**

Supporting Information

Spatiotemporal Immunomodulation and Biphasic Osteo-Vascular Aligned Electrospun Membrane for Diabetic Periosteum Regeneration

Yusen Qiao, Lei Yu, Peng Yang, Miao Chen, Haifu Sun, Lingjie Wang, Bangzhao Wu, Chun-do Oh*, Huilin Yang*, Jiaxiang Bai*, Dechun Geng**

Dr. Y. Qiao, Dr. L. Yu, Dr. P. Yang, Dr. M. Chen, Dr. H. Sun, Dr. L. Wang, Dr. B. Wu, Dr. J. Bai, Prof. Dr. H. Yang, Prof. D. Geng
Department of Orthopedics, The First Affiliated Hospital of Soochow University, 188 Shizi Road, Suzhou 215006, Jiangsu, China
E-mail: hlyang@suda.edu.cn (H. Yang); szgengdc@suda.edu.cn (D. Geng)

Prof. Dr. J. Bai
Department of Orthopedics, The First Affiliated Hospital of USTC, Department of Orthopedics, Centre for Leading Medicine and Advanced Technologies of IHM, The First Affiliated Hospital of USTC, Division of Life Sciences and Medicine, Hefei, 230022, China. National Center for Translational Medicine (Shanghai) SHU Branch,Shanghai, China
E-mail: 20174232033@stu.suda.edu.cn (J. Bai)

Dr. Y. Qiao, Prof. CD. Oh
Department of Orthopedic Surgery, Rush University Medical Center, Chicago, IL 60612, USA.

Experimental Section

Materials: PLA (Mw = 109–150 kDa, IV= 4.0-5.0) was obtained from Jinan Daigang (Jinan Daigang Co., China). Rat tail COLI was purchased from Sigma Aldrich (USA). Dulbecco's Modified Eagle Medium (DMEM), fetal bovine serum (FBS), trypsin, and penicillin/streptomycin were purchased from Gibco (USA).

Preparation of the aligned nanofiber scaffolds: PLA (0.5 g) was dissolved in dichloromethane (4.0 g, DCM, Sinopharm Reagent Co., Beijing, China) and N, N, DMF (2.0 g Shanghai Lingfeng, Shanghai, China) solution at room temperature (25 ºC). The electrospun solution was inserted into a syringe measuring 10 cm in length and 0.9 mm in diameter for the preparation of fiber scaffolds with different orientations. Electrospinning had a propulsion pump speed of 70 µL min^{-1}, at 18 kV, with a distance of 15 cm between the tip of the needle and the parallel electrode receiver. Parallel oriented fiber scaffolds were collected between the electrode rods.

Preparation of glucose-responsive liposomes: The glucose-responsive liposomes were prepared by reverse evaporation. More specifically, lecithin (160 mg, Shanghai Yuanye, China), cholesterol (40 mg, Arcos, Belgium), and DSPE-PEG2K-FPBA (4 mg, Ruixi, Shanxi, China) were dissolved in trichloromethane (5 ml, Shanghai Lingfeng, Shanghai, China). The resulting solution was then mixed with trichloroethane (1 ml), containing octadecylamine (5 mg, Shanghai Aladdin Chemical Co., Ltd., China) and APY29 (4 mg, Shanghai MCE, China). The solution was further ultrasonically mixed to obtain a uniform emulsion. The colloidal product was obtained by removing organic solvents with a rotary evaporator. Finally, a hydration treatment was carried out to prepare the liposome emulsion. The liposomes were obtained by ultrasound and filtration using 450 nm and 220 nm polycarbonate membranes (Millex-GP, Ireland). Blank liposomes were prepared from lecithin, cholesterol, octadecylamine, and DSPE-PEG2K-FPBA (40:10:5:4, w/w/w/w). All liposomes were stored after lyophilization.

Construction of the functional membranes: Prior to functionalization of the PLA membranes with COLI and the prepared liposomes, the membranes were sterilized with 75% ethanol for 30 min, followed by rinsing with deionized water three times to remove any residual ethanol. Lyophilized COLI was dissolved in an aqueous solution of acetic acid (0.1 M, 3 mg ml^{-1}) and stored at 4 °C until further use. In an ice bath, the diluted COLI solution was further diluted with PBS (10×) at a ratio of 6:1, and the pH was adjusted to 7.0 with an aqueous NaOH (0.1 M) solution. The liposomes (15.6 µg) were then added to a neutralizing collagen solution (100 µL), stirred thoroughly, and placed on the electrospun membranes. Finally, the samples were placed in incubators at 37 ºC for 30 min for self-assembly. The samples were then washed three times

with deionized water to obtain the desired PCLA membranes, which were immediately used for material characterization or cell experiments.

Characterization: SEM was performed using a Hitachi S-4800 scanning electron microscope (Japan). The average diameter of 200 random fibers was measured with ImageJ.[36] The orientations of the fiber scaffolds were determined using the orientation plugin of ImageJ, while the structures of the samples were imaged using a confocal microscope (Axio Imager M1, Zeiss, Germany). The FTIR spectra were obtained on Frontier TM Fourier transform infrared spectrometer (PerkinElmer, USA). Rheological tests and water contact angle measurements were performed using a HAAKE RheoStress 6000 instrument (USA Thermal Sciences) and the contact angle meter (Data Physics Corporation), respectively. Mechanical properties and nanofiber surface elements were investigated using the General Dynamics Test System (Hengyi, Shanghai, China) and XPS System (250Xi, American USA Scientific Escalab), respectively. The topological morphology of the fiber surfaces was observed using AFM (Dimension ICON, Bruker, USA), while the surface morphology of liposomes was observed using TEM (HT7700, Hitachi, Japan). The particle size, PDI, and potentiodynamic potential of each mixture were measured using the dynamic light scattering granularity analyzer (Nano-ZS 90 Zeta sizer, Malvern, UK). To measure the release of APY29 from the liposomes at different glucose concentrations (25, 5.6, and 0 mM), the liposomes were immersed in 50 mL centrifuge tubes containing the corresponding glucose solution (10 mL) for 3, 6, 9, 12, 15, 18, 21, and 24 h. The degree of APY29 release was determined using HPLC (HPLC, Agilent system with Kromasil 100-5C18 column), and the corresponding cumulative release curves were plotted.

Preparation of the cell cultures: The RAW264.7 cells were cultured in DMEM (Hy Clone) at 37 °C, and the BMSCs were cultured in α-MEM with 5% CO_2, both containing 10% FBS (Gibco) and 1% penicillin/streptomycin. Before cell culture, the hybrids were disinfected overnight with ethanol under UV light and then washed with disinfected PBS.

Cell viability and proliferation: The survival rates of the BMSCs and RAW264.7 cells on the different fiber membranes were evaluated by staining with a live-dead cell staining kit (Invitrogen, USA) after culturing for 3 d. The stained cells were observed under a fluorescence microscope (Axio Imager M1, Zeiss, Germany), and semi-quantitative fluorescence analysis was performed using ImageJ. Cell counting kit-8 (NCMBio, Suzhou, China) was used to study the proliferation rate of BMSCs on cell membranes 3 and 7 d after transplantation. More specifically, the BMSCs (5×10^3 cells/well) were implanted in a 96-well plate (100 μg per well).

On days 3 and 7, the absorption of each plate was measured with a microplate reader at 450 nm of light density (Biotek, USA).

Cell adhesion: The BMSCs (1×10^4 cells/well) were seeded on cell creepers in the hybrid material extracts. After one day of co-culture in a 24-well culture dish, the cells were immobilized on ice for 30 min with 4% paraformaldehyde, and the membrane was perforated for 10 min with a solution containing 0.1% Triton X-100 (Sigma Aldrich, USA). To avoid nonspecific staining, bovine serum albumin was used to block cells overnight at 4 ^{O}C. The cells in the medium were then fixed with 4% paraformaldehyde and incubated at 4 ^{O}C at night with a primary vinculin antibody (ab130007, Abcam). Subsequently, the cells were washed with 1 PBS containing 0.1% Tween to remove any unconjugated primary antibodies, and then incubated with a fluorescent secondary antibody (ab150115, Abcam) for 1 h. FITC phalloidin was subsequently added to stain the F-actin, while 4′ ,6′ -diamidino-2 -phenylindole (DAPI) was added to stain the nuclei. Fluorescence images were obtained under laser excitation using a fluorescence microscope (Axio Imager M1, Zeiss, Germany).

Cell morphology: Membranes were cultured with BMSCs (2×10^4 cells/well) for 3 d and immobilized with 4% paraformaldehyde on ice for 1 h. Subsequently, the cells were washed with aqueous ethanol solutions of different concentrations (30, 50, 70, 80, 90, 95, 100, and 100%) for 15 min each. After approximately 2 h of drying with a CO_2 cutoff dryer, the sample surface was gilded for 45 s, rinsed 3 times with PBS, and fixed with polyformaldehyde for 30 min before SEM images were obtained.

In vitro osteogenesis: The osteogenic ability of the prepared materials was evaluated using the alkaline phosphatase assay kit (Beyotime Biotechnology, Shanghai, China). More specifically, BMSCs (1×10^4 cells/well) were seeded in 24-well plates using a mixture extracted from different populations. After 7 d, the cells were fixed with 4% paraformaldehyde. Next, an alkaline phosphatase staining solution (300 μL/well) was added to each well in the absence of light, and allowed to stand for 30 min. The stained (positive) cells were visible under an optical microscope. After 2 d of culture, the cells were immobilized in 4% paraformaldehyde for 30 min. Subsequently, the cells were cleaned several times with PBS to remove the excess dye, and the calcium nodules were observed with an optical microscope. To quantify the ARS staining results, 5% perchloric acid was added to each well and uptake was measured at 490 nm (Bio-Rad 680).

Immunofluorescence: Initially, the BMSCs were washed three times with PBS and incubated in cold paraformaldehyde for 15 min. The BMSCs were then blocked with QuickBlockTM IF

blocking solution (Beyotime Biotech) and cultured at 4 °C for 1 h, followed by primary antibodies overnight at 4 °C. After two subsequent PBS washes, the cells were cultured at room temperature (25°C) with IgG H&L antibodies (Alexa Fluor 647;Abcam, ab150079; Alexa Fluor 488; Abcam, ab150165) for 1 h. Finally, the BMSCs were stained with DAPI and intracellular protein expression was observed through confocal fluorescence microscopy.

In vitro angiogenesis: Growth factor reductant substrate (Matrigel 100 μg/well, Corning, USA) was employed to observe the angiogenic capabilities in different material groups. HUVECs (3.5×10^4 cells/well) were then seeded into each well of the mixture. After incubation at 37 °C in an atmosphere containing 5% CO_2, each plate was observed with an optical microscope after 0, 3, and 6 h. The migration ability was verified using Transwell plates (Corning). Specifically, PLA, PC, PCL, and PCLA extracts were placed in lower chambers, while the HUVEC suspension (200 μL, 2.5×10^5 cells ml^{-1}) was added to the upper chambers and incubated at 37 °C in an atmosphere containing 5% CO_2. After 16 h of culture, the plates were removed from the incubator was , the upper membrane was carefully removed with cotton swabs and immobilized with 4% paraformaldehyde for 20 min. The cells were then stained with 0.1% crystal violet solution (Solarbio, Beijing, China) and then observed under an optical microscope. Wound healing was assessed to evaluate angiogenesis and 4×10^5 cells/well were seeded in a 6-well plate and incubated at 37 °C in an atmosphere containing 5% CO_2. When the flow of the cells reached 90% fluency, scratches were made in the cell layer using the tip of a 200 μL pipette. All images were captured with inverted microscopes after 0, 12, and 24 h.

Identification of the macrophage phenotype: Polarized RAW264.7 cells were altered at gene expression levels under the influence of reactive fibrillar membrane. The main antibodies, iNOS (M1 marker) and Arg-1 (M2 marker) containing F-actin, were then incubated with the cells and stored overnight at 4 ºC. The cells were cultured with IgG H&L (Alexa Fluor 647, Red; Abcam; Alexa Fluor 488, Green; Abcam) and DAPI solution. All images were captured with fluorescent microscopes. On day 7 of co-culture, the M1 and M2 macrophages were treated with iNOS (Abcam, ab49999, 1:100) and Arg-1 (Abcam, ab239731, 1:100), and later incubated with goat anti-rabbit (Abcam, ab155079, 1:200) and goat anti-mouse (Abcam, ab150113, 1:400). Semi-quantitative analysis was then performed using ImageJ. Different groups of cell suspensions were centrifuged for 10 min at 300 g, and antibodies were added for 30 min before further incubation at 4 ºC. For this purpose, the following murine conjugated antibodies were used: mouse anti-CD11b-APC (562102, 1:20; BD Pharmingen), mouse anti-CD86-FITC (561961, 1:20; BD Pharmingen), and mouse anti-CD206-PE (sc-58986, 1:50; Santa Cruz Biotechnology). The cells were then analyzed using a flow cytometer (Merck Millipore, USA), and the results

were analyzed using FlowJo 7.6. To this purpose, cells were initially subjected to CD11b gate control to ensure that only myeloid cells were selected, and a combination of specific markers was then used to identify M1 (CD86) and M2 (CD206) macrophages. Three samples were randomly selected from each group (n = 6).

RT-PCR: Total RNA was isolated from the BMSCs according to the standard protocol. The mRNA concentrations and purity were assessed using NanoDrop-2000 (Thermo Fisher Scientific, Waltham, MA, USA). The mRNA expression was calculated using the $2^{-\Delta\Delta Cq}$ method.[37] Primer sequences are shown in Table S2.

Preparation of the conditioned media: A combination of conditioned media was collected to induce osteogenesis and angiogenesis. To this end, RAW264.7 cells (4×10^5 cells/well) were cultured on 6-well plates in the atmosphere containing 5% CO_2. Each culture medium was treated with LPS (100 ng mL^{-1}) for 12 h after culture, followed by collection and filtration of different groups of the supernatants to remove cellular debris in an aseptic environment.

Animal models: Female Sprague-Dawleyrats (average weight: 200–250 g) were purchased from the Soochow University Laboratory Animal Centre (ethics approval number: 20220925A01). Rats were given an intraperitoneal injection with STZ (35 mg kg^{-1}). Blood was extracted and stored weekly from the tail vein to analyze blood sugar levels, with values > 16.7 mm being considered those of a successful diabetes model. The rats were then intraperitoneally anesthetized with 2% pentobarbital sodium. After complete shaving and disinfection, a longitudinal incision was made in the middle of the surgical area, carefully separating the soft tissue to expose the calvarium. The periosteum was removed, and two bilateral defects measuring 5 mm in diameter were carefully created on the skull using a dental trephine to simulate poor bone conditions and periosteal defects. The defect area was then covered with a thin film. Penicillin was injected once daily for a period of 3 d.

Micro-CT assessment: SD rats were euthanized 4 and 8 weeks after surgery, and calvarial specimens were collected and immobilized in 10% formalin for further identification. Micro-CT (SkyScan 1176, SkyScan, Belgium) was originally used to assess regeneration conditions in defective areas using settings such as 65 kV, 385 mA, and 1-mm Al filters. The skulls were reconstructed in 3D using the Mimics software (version 21.0). For calculating BV/TV, BMD, Tb.N, and Tb.Sp, a cylindrical space representing the region of interest was designated to assess bone and tissue volumes. The values were obtained using CT Analyzer software (SkyScan, Belgium).

Histological analysis: 2, 4, and 8 weeks after surgery, each calvarium was removed and fixed/decalcified at room temperature (25℃) with 10% formic acid for 1 week, followed by alcohol gradient dehydration and paraffin blocks. The embedded specimens were sliced into 6-μm thick histological sections at the center of the defect zone and the morphological changes were observed under an optical microscope using conventional routine H&E and Masson staining (Carl Zeiss). Levels of iNOS, CD31, Runx2, and periostin markers were also identified by immunohistochemistry staining. More specifically, the slices were dewaxed and gradient hydrated for 30 min at 37 °C before being treated with trypsin. Diluted horse serum was added after 30 mins to block any nonspecific sites and then three PBS purges were performed. Subsequently, major antibodies were added and incubated overnight at 4 °C. The slices were then cultured for 30 mins with corresponding secondary and tertiary antibodies.

Statistical analysis: Data were presented as means ± standard deviations. Unless otherwise noted, statistical analysis (Origin 9.1 or GraphPad Prism 7.0 software) through a one- or two-way analysis of variance was done to evaluate the differences between groups using Tukey's multiple comparison test. A p-value < 0.05 was considered statistically significant.

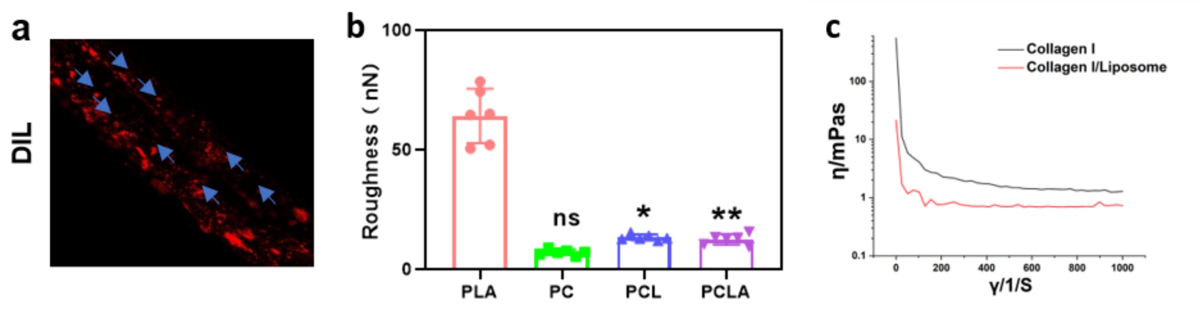

Figure S1. Physicochemical properties and morphology of composites. (a) Cell membrane red fluorescent probe (Dil) of liposomes deposited on the spinning surface. (b) Roughness of PLA, PC, PCL, and PCLA surfaces. (c) Rheological test of PLA, PC, PCL, and PCLA. (n = 6 per group, data are provided as means ± SD, *p < 0.05, **p < 0.01, ns, no significance).

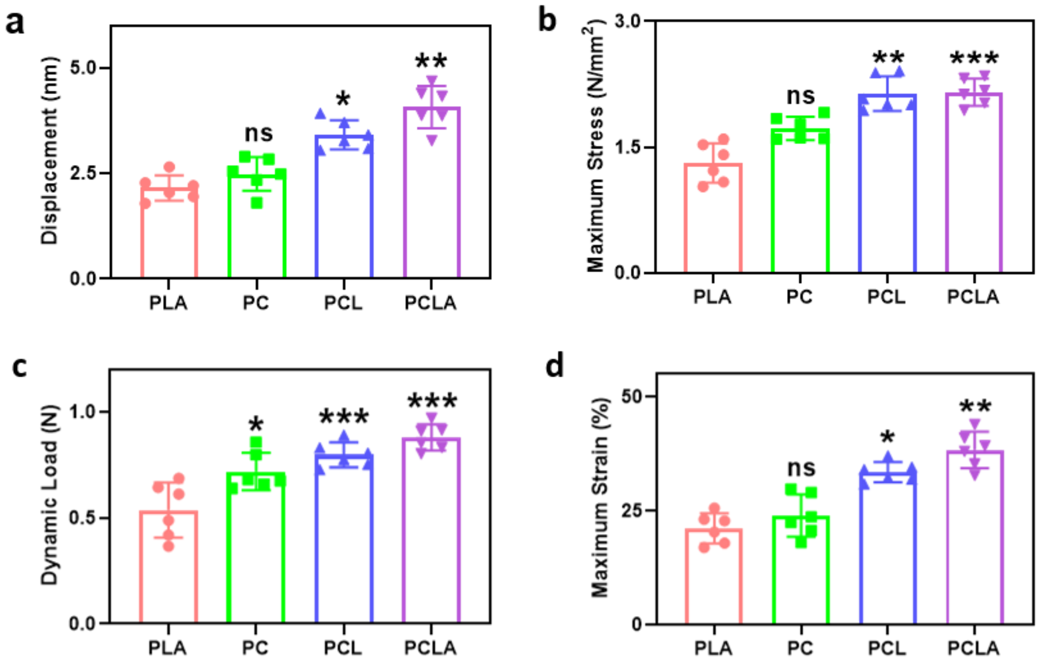

Figure S2. Mechanical characteristics of PLA, PC, PCL, and PCLA. (A) Displacement. (B) Maximum Stress. (C) Dynamic load. (D) Maximum Strain. (n = 6 per group, data are provided as means ± SD, $*p < 0.05$, $**p < 0.01$, $***p < 0.001$, ns, no significance).

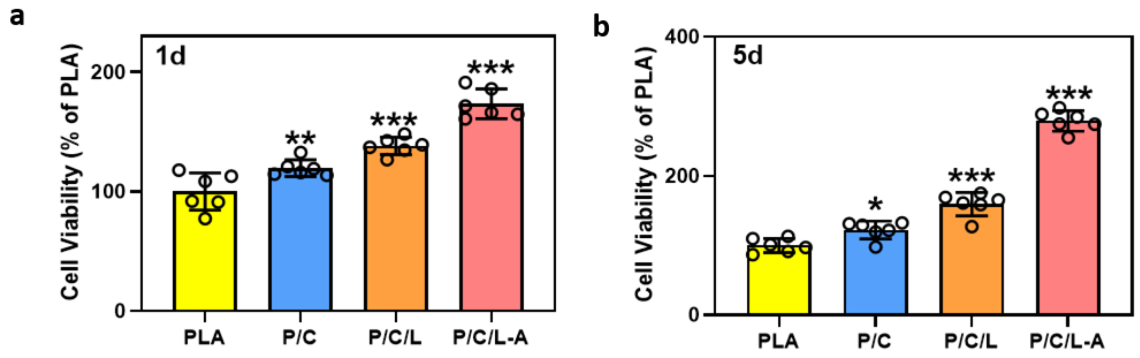

Figure S3. CCK-8 assay of BMSC cells in hydrogel extracts for 1 and 5 d. (a) Day 1 and (b) day 5 (n = 6 per group, data are provided as means ± SD, $^{*}p < 0.05$, $^{**}p < 0.01$, $^{***}p < 0.001$).

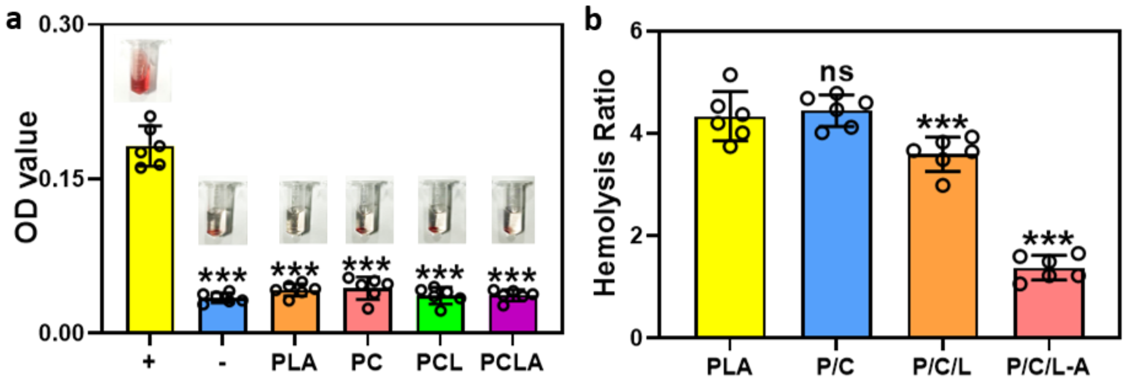

Figure S4. Blood compatibility of PLA, PC, PCL, and PCLA. (a) General view. (b) OD value and (c) hemolysis ratio (n = 6 per group, data are provided as means ± SD, ***$p < 0.001$, ns, no significance).

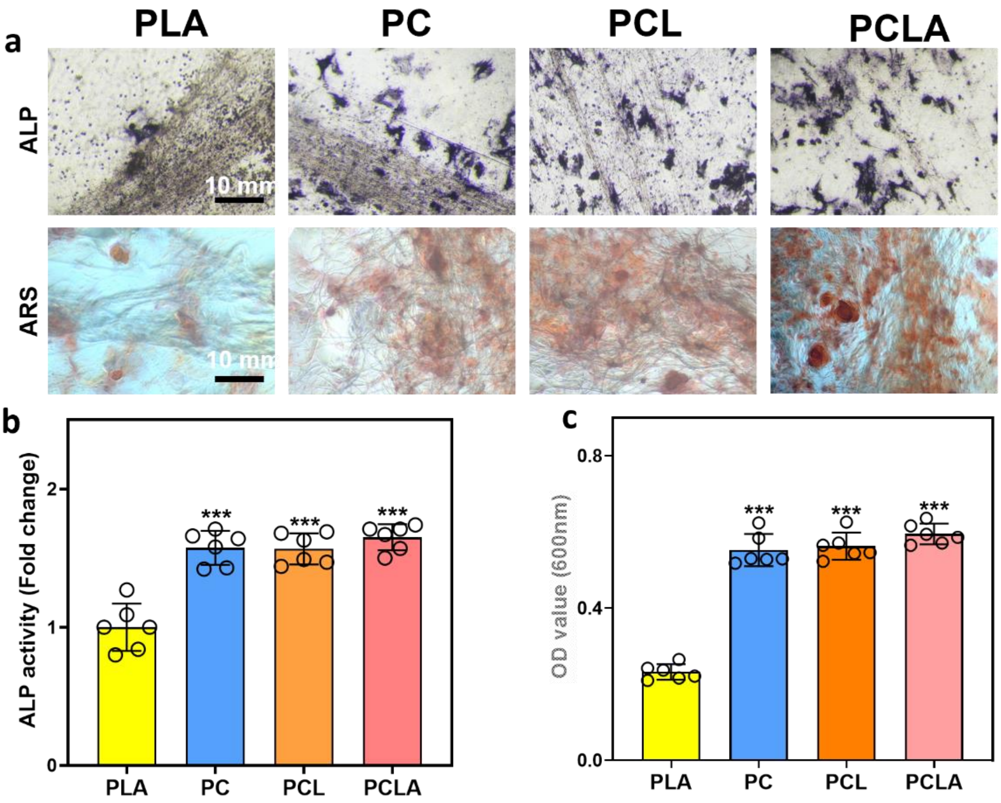

Figure S5. Synergetic effects of PLA, PC, PCL, and PCLA on osteogenesis induction in vitro. (a) ALP and ARS staining at 7 and 21 days using BMSC cells. (b) Quantitative evaluation of ALP activity. (c) Quantification of ARS at OD value (490 nm).

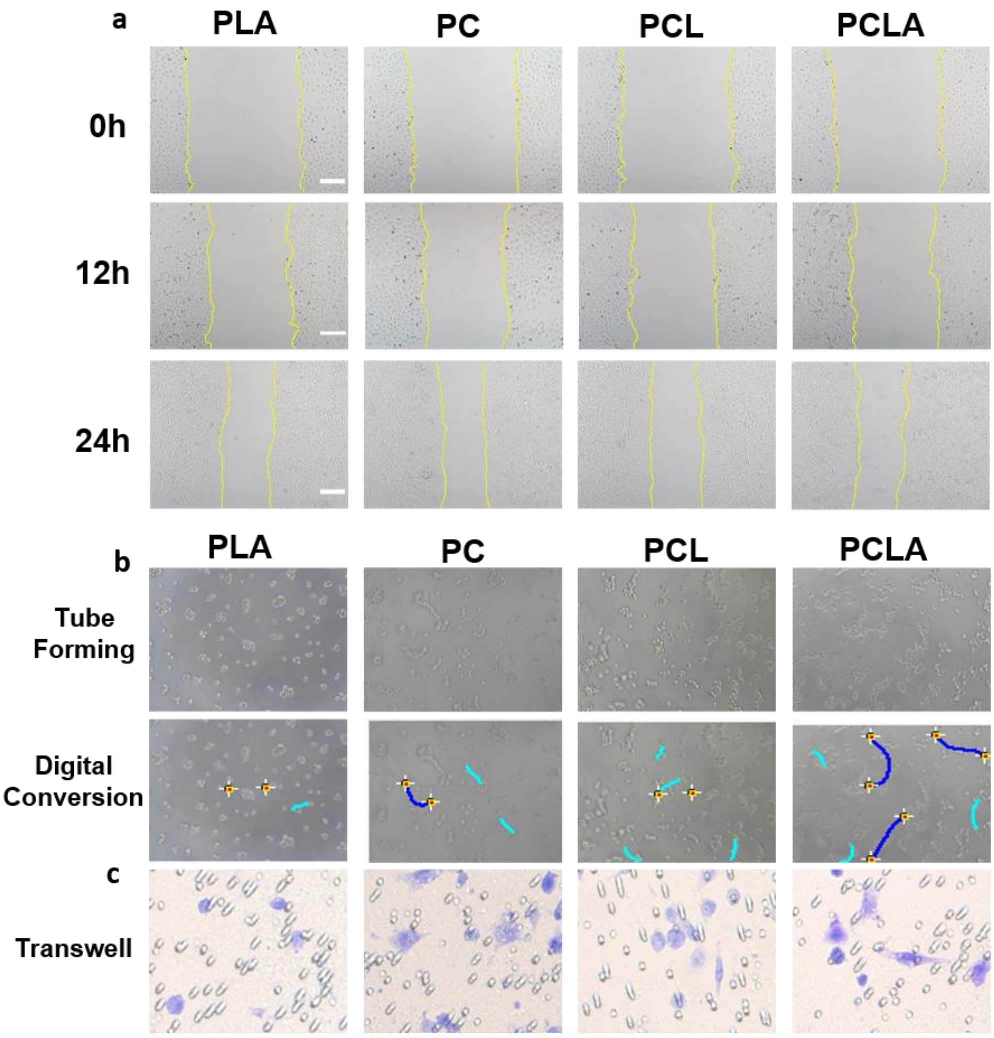

Figure S6. Synergetic effects of PLA, PC, PCL, and PCLA on angiogenesis in vitro. (A) Wound healing assay (scale bar: 200 µm). (B) Tube forming assay (scale bar: 100 µm). (C) Migration assay (scale bar: 200 µm).

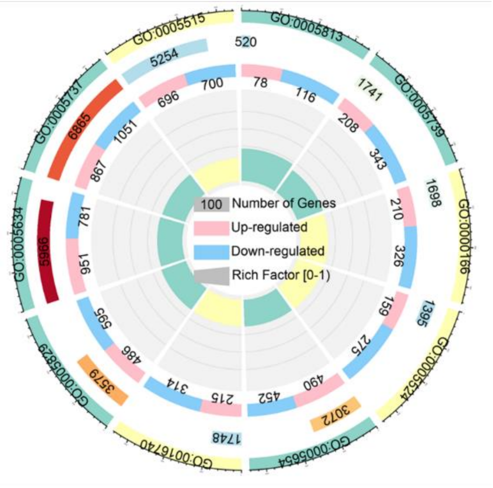

Figure S7. GO enrichment analysis circle of differentially expressed genes in PCLA and DM groups.

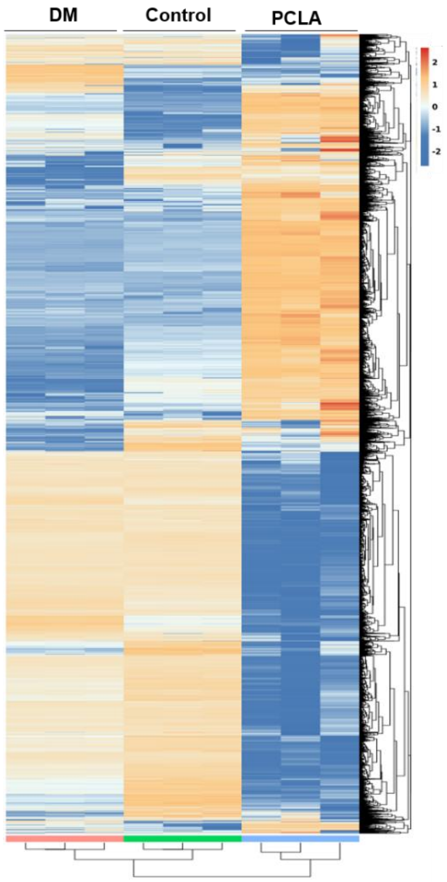

Figure S8. Cluster map of differential gene grouping (Control, DM, and PCLA).

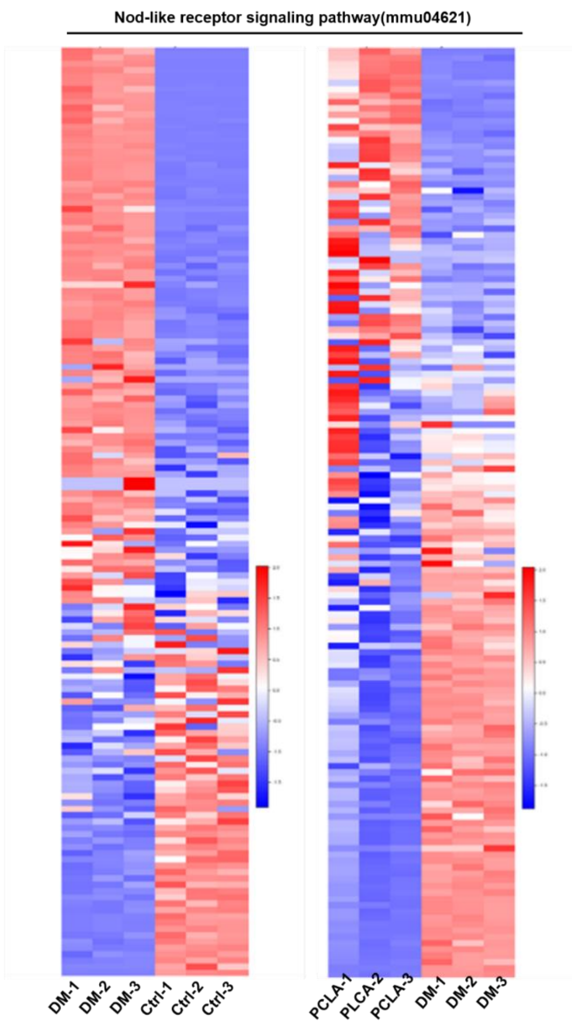

Figure S9. Differential expression of inflammation related biomarkers in NOD-like receptor signaling pathways in the osteogenic-induced MC3T3-E1 cells of the DM, Control, and PCLA groups (n = 3). Pro-inflammatory genes—red dashed lines; anti-inflammatory genes—blue dashed lines.

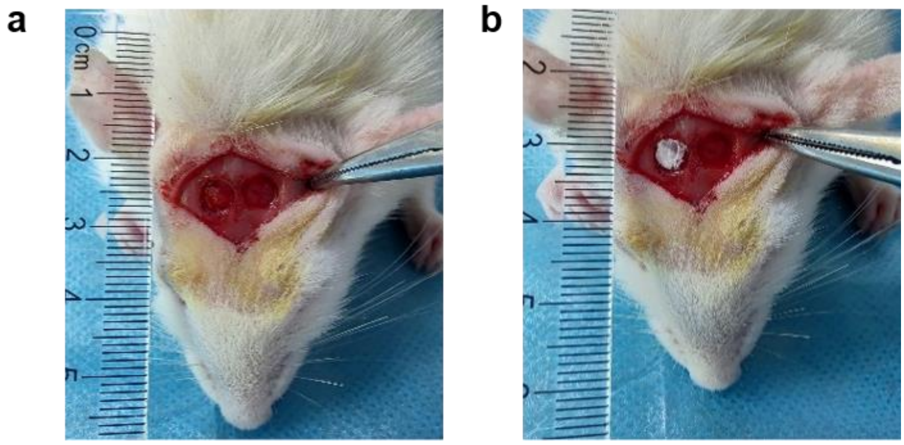

Figure S10. Animal Skull defect modeling.

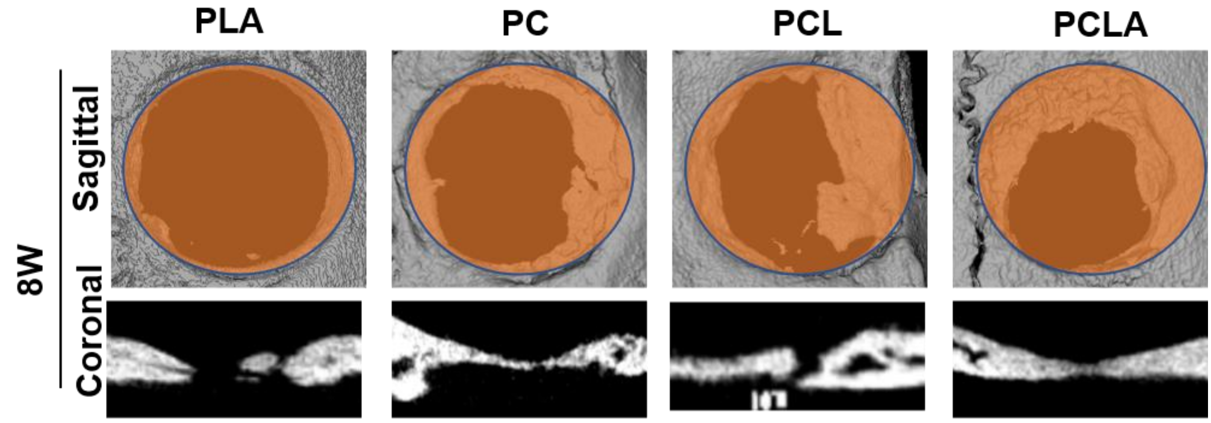

Figure S11. Micro-CT evaluation of PLA, PC, PCL, and PCLA (n = 6) after implantation of composites (8 weeks).

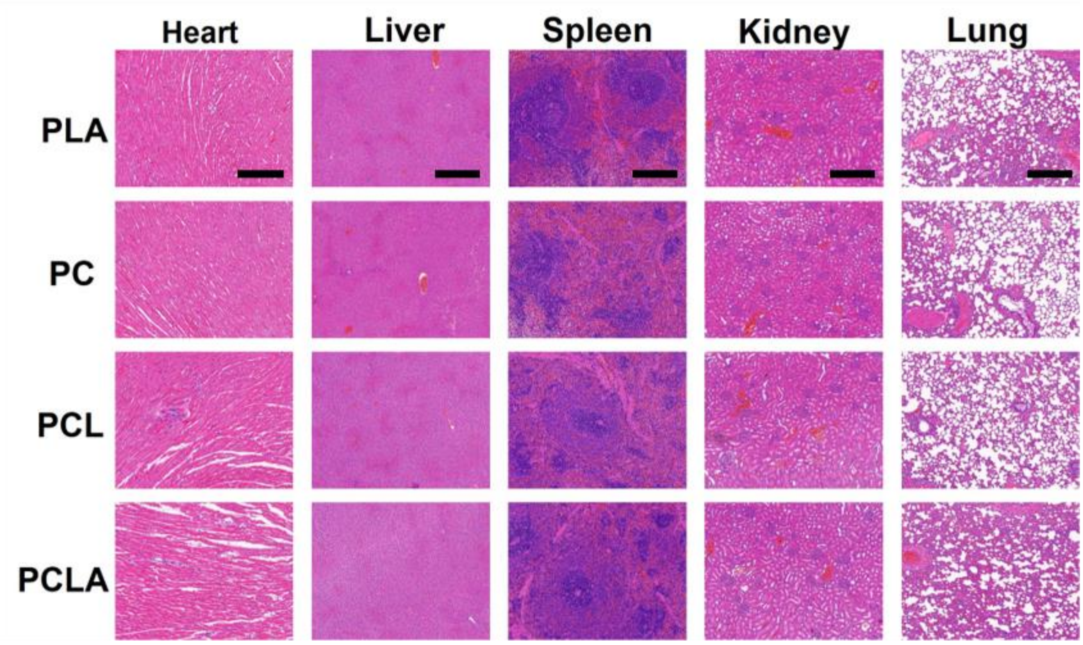

Figure S12. H&E staining of PLA, PC, PCL, and PCLA groups at 8 weeks: heart, liver, spleen, kidney, and lung (scale bar: 200µm).

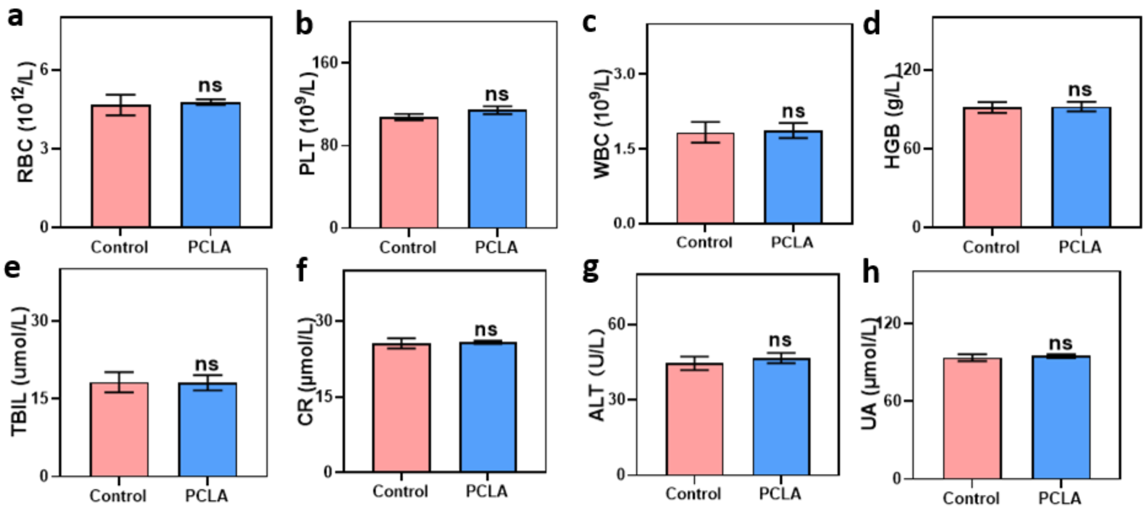

Figure S13. Biosafety evaluation of control and PCLA. (A) TBIL, (B)PLT, (C)RBC, (D) HGB, (E) ALT, (F) CR, (G) UA, and (H) WBC (n = 2 per group, data are provided as means ± SD, ns, no significant).

Table S1 Material grouping

Group	Denoted	Function
PLA electrospinning	PLA	Control
Collagen self-assembled on PLA electrospinning	PC	Control
Collagen self-assembled on PLA electrospinning carrying blank liposomes	PCL	Control
Collagen self-assembled on PLA electrospinning carrying liposomes loading APY29	PCLA	Test

Table S2 Primers for RT-PCR on osteogenesis, angiogenesis and anti-inflammation

Mouse

Gene	Forward	Reverse
β-actin	*GTGCTATGTTGCTCTAGACTTCG*	*ATGCCACAGGATTCCATACC*
IL-1β	*TGGAGAGTGTGGATCCCAAG*	*GGTGCTGATGTACCAGTTGG*
BMP-2	*GCTCCACAAACGAGAAAAGC*	*AGCAAGGGGAAAAGGACACT*
VEGF-A	*GTCCCATGAAGTGATCAAGTTC*	*TCTGCATGGTGATGTTGCTCTCTG*
IL-10	*GAGAAGCATGGCCCAGAAATC*	*GAGAAATCGATGACAGCGCC*

Rat

Gene	Forward	Reverse
GAPDH	*ACTCCCATTCTTCCACCTTTG*	*CCCTGTTGCTGTAGCCATATT*
BMP-2	*AACGAGAAAAGCGTCAAGCC*	*AGGTGCCACGATCCAGTCAT*
Osterix	*GCCTACTTACCCGTCTGACTTTGC*	*CCCTCCAGTTGCCCACTATTGC*
Runx2	*TCACAAATCCTCCCCAAGTGG*	*GAATGCGCCCTAAATCACTGA*

Human

Gene	Forward	Reverse
GAPDH	*CGGAGTCAACGGATTTGGTCGTAT*	*AGCCTTCTCCATGGTGGTGAAGAC*
VEGF-A	*TTAAACGAACGTACTTGCAGATG*	*GAGAGATCTGGTTCCCGAAA*
